# Actions of Midostaurin as Cation Channel and Tyrosine Kinase Inhibitor in Diffuse Intrinsic Pontine Glioma Cell Lines

**DOI:** 10.3390/cancers18071066

**Published:** 2026-03-25

**Authors:** Marina Antonacci, Annamaria Di Turi, Morena Miciaccia, Michele Denora, Fatima Maqoud, Maria Grazia Perrone, Antonio Scilimati, Domenico Tricarico

**Affiliations:** 1 Department of Pharmacy-Pharmaceutical Sciences, University of Bari Aldo Moro, 70125 Bari, Italy; marina.antonacci@uniba.it (M.A.); a.dituri6@phd.uniba.it (A.D.T.); morena.miciaccia@uniba.it (M.M.); m.denora10@alumni.uniba.it (M.D.); mariagrazia.perrone@uniba.it (M.G.P.); 2 Functional Gastrointestinal Disorders Research Group, National Institute of Gastroenterology Saverio de Bellis, Istituti di Ricovero e Cura a Carattere Scientifico (I.R.C.C.S.) Research Hospital, 70013 Castellana Grotte, Italy; f.maqoud@gmail.com

**Keywords:** midostaurin, antiproliferative effect, diffuse intrinsic pontine glioma cell lines, tyrosine kinase, ion channels, patch clamp

## Abstract

Tyrosine kinases (TKs) are drug targets in diffuse intrinsic pontine glioma (DIPG). Ion channels are emerging targets in cancer. TKIs targeting different kinases such as everolimus, crizotinib, dasatinib, erlotinib, lapatinib, perifosine and midostaurin (0.001–100 μM) were investigated on cell proliferation and ion channel currents. **Methods**: Cell viability assays in parallel with a patch-clamp study and Western blot of target proteins are performed in SU-DIPG-36 and SU-DIPG-50 cells. **Results**: Midostaurin is the most effective drug in different assays. Patch-clamp investigations show that the application of midostaurin reduced the inward and outward whole-cell cation channel currents vs. controls in the presence of low internal ATP. These currents were sensitive to the KATP channel inhibitors glibenclamide and repaglinide and were fully reduced by the unselective blocker TEA-BaCl_2_. Midostaurin also reduced currents that are sensitive to TRPV1 channel blockers capsazepine and ruthenium-red. The IC_50_ values of midostaurin as an antiproliferative drug and ion channel inhibitor in either cell line are in the sub-micromolar range. In SU-DIPG-36 cells midostaurin causes a concentration-dependent upregulation of autophagy markers. **Conclusions**: The inhibition of cation channel currents by midostaurin in SU-DIPG-36 and SU-DIPG-50 cells and the autophagy potentiation in SU-DIPG-36 cells can be novel mechanisms in DIPG.

## Simple Summary

Diffuse intrinsic pontine glioma (DIPG) is a pediatric high-grade glioma which has no effective treatment to date and for this reason has a fatal outcome. SU-DIPG-36 are H3.1K27M mutated cells and SU-DIPG-50 are H3.3K27M mutated. H3K27M mutations frequently co-occur with alterations in key signaling pathways, involving extracellular receptor tyrosine kinases (KIT, MET, EGFR, PDGFR, VEGFR), cyclin-dependent kinases (CDK1, CDK4, CDK6), the activin A receptor (ACVR1), transcriptional regulators such as MYCN, tumor suppressor genes including PTEN and TP53, as well as intracellular kinases of the PI3K/AKT/mTOR pathway. The aim of this scientific work, since tyrosine kinases are molecular targets in DIPG, is to investigate several tyrosine kinase inhibitors. Some tyrosine kinases regulate ion channels and transporters, and ion channels are emerging targets in cancer. This study is pursued through cytotoxicity assays, characterization of ion channel currents and Western blot analysis on target proteins.

## 1. Introduction

The treatment of diffuse midline glioma (DMG) which is a fatal pediatric high-grade glioma (pHGG) is a global challenge [1,2,3]. DMG is mostly diagnosed in the midline of the brain, especially in the pons, and historically referred to as diffuse intrinsic pontine glioma (DIPG/DMG) and represents 10–15% of all pediatric brain tumors. DIPG patients face an extremely poor median overall survival (OS) up to 12 months and about 90% of children die within two years of initial diagnosis and less than 1% survive after 5 years [4,5]. In recent decades, distinct pHGG entities have been identified based on recurrent epigenetic alterations [6].

Tyrosine kinases have been proposed as molecular targets in DIPG. The H3.1 or H3.3 variants (HIST1H3B/C and H3F3A) of histone H3, altering the epigenetic landscape of precursor astrocyte or primitive oligodendrocyte cells of the pontine region of the brainstem, are the common somatic mutations in this disease. H3K27M co-occur with alterations in the cascade of signaling genes, including extracellular receptor tyrosine kinases (KIT/MET/EGFR/PDGFR/VEGFR), cyclin-dependent kinases (CDKs1/4/6), activin A receptor (ACVR1), transcriptional regulators (MYCN) and tumor suppressors (PTEN/TP53), and intracellular kinases (PI_3_K/AKT/mTOR) [7]. Among those, mTOR emerged as a targetable molecular pathway across DIPG patient models, using blood–brain-barrier-penetrant PI3K/Akt/mTOR inhibitors like paxalisib. This drug potentiates calcium-activated PKC signaling and, in combination with the brain-penetrant protein kinase C (PKCb) inhibitor enzastaurin, prolonged the survival of multiple orthotopic patient-derived and immunocompetent allograft models [8].

Ion channels and transporters are upregulated in 33% and downregulated in 48% of cases of pediatric brain tumor and can be novel targets in cancer [9]. Ion channels and transporters have a role in cell division [10] and are increasingly considered to be of pathophysiological relevance in tumor biology [11,12]. Although these proteins are inherent in brain functioning (neuronal and glial) and dysregulated in brain tumors [13,14,15], currently no data is available on genes encoding ion channels and transporters and their relevance in DIPG.

We recently found that repaglinide is very effective in reducing cell proliferation and apoptosis in SU-DIPG-36 and SU-DIPG-50 cells and the effects were associated with histone deacetylase modulation; this makes it an interesting candidate to study, as well as its known action on KATP channels, where it acts as a blocker of ATP-dependent potassium channels [16]. The KATP-ATP-sensitive potassium channel is a type of potassium channel that is gated by intracellular nucleotides, ATP and ADP. The channel is an octameric complex of inwardly rectifying potassium channel family Kir6.1 and Kir6.2 subunits and of sulfonylurea receptor type 1 (SUR1) and type 2 (SUR2). These channels are specifically modulated by class of drugs known as KATP channel openers like diazoxide and inhibited by sulfonylureas repaglinide and glibenclamide [17,18,19]. A high expression of the *ABCC8* gene encoding for the SUR1 subunit has been reported in patients with glioma with a higher survival rate and response to temozolomide [20] and in glioma cells regulating ERK [21]. The well-known reference compound of the PKC inhibitors staurosporine reduces KATP channel currents in the absence of intracellular ATP while inducing activation of the channel current in the presence of nucleotide and chloride conductance in skeletal muscle fibers [22,23]. PKC enzymes are well-known regulators of ion channels, including KATP and TRPV1 channels in different cells.

In our experiments we also found capsaicin and capsazepine-sensitive ion channel currents in SU-DIPG-36 and SU-DIPG-50 cells that are known, respectively, as agonists and antagonists of transient receptor (TRP) channels [16]. These ion channels mostly carry Ca^2+^ ions with a role in cell proliferation, apoptosis, and resistant mechanisms. The downregulation of the gene expression of the family members *TRPM3* (*melastatin 3*)*, TRPV1* (*vanilloid 1*)*, TRPV2* (*vanilloid 2*), and *TRPML1* (*mucolipin 1*) has been found inversely correlated with tumor grade increases, suggesting them as prognostic markers for glioblastoma patients [24]. In particular, *TRPV1_V_*_3_ variant mRNA is correlated with the survival rate of these patients [25]. Transient receptor potential cation channel subfamily V member 1 (TRPV1) is also known as the capsaicin receptor, a member of the TRPV group of the TRP channels that are drug targets [26]. The function of TRPV1 is detection and regulation of body temperature and analgesic response to thermal stimulus [27]. In this respect, we found that the TRPV1 channel agonist capsaicin at 1 µM and the antagonist capsazepine at 1 µM caused, respectively, +26% and −15% changes in cell proliferation after 6 h of incubation in SU-DIPG-36 cells. Higher concentrations of 20 µM capsaicin caused a mild reduction in cell proliferation of −17% in this cell after 48 h of incubation [16]. In DIPG-50 cells, the TRPV1 agonist capsaicin reduced cell survival by −35% after 72 h of incubation while capsazepine was not effective [16], in line with the positive prognostic role of the TRPV1 gene observed in glioblastoma [24,25].

Here, we describe a pharmacological investigation conducted on two patient autopsy-derived DIPG cell cultures (SU-DIPG-36, SU-DIPG-50) (Dr Michelle Monje). Specifically, SU-DIPG-36 cells are H3.1K27M mutated cells and SU-DIPG-50 are H3.3K27M mutated cells selected as representatives of diverse DIPG pathological characteristics and conditions. Specifically, they respond differently to ONC201 [2] and are characterized by the predominant molecular profile (SU-DIPG-36 has H3.1K27M, whereas SU-DIPG-50 has H3.3K27M) of patients diagnosed with DIPG. In addition, these two cell lines derived from DIPG patients were selected to investigate unexplored DIPG biochemical pathways to identify new therapeutic strategies for the treatment of DIPG. The effects of TKIs on SU-DIPG cells were studied in 96-well crystal violet, CCK-8 assays, and clonogenic assays. The most effective TKI found in cell proliferation experiments was tested on ion channel currents by patch clamp. The responses of the TKI-sensitive currents to KATP channel inhibitors such as glibenclamide, repaglinide and Ba^2+^ ions and to TRP inhibitors such as ruthenium red and capsazepine were evaluated in the same cells. The effects of the most effective TKI in different cell proliferation assays on cell death signaling and target proteins were investigated by Western blot in these cells. The selected TKIs were those targeting different tyrosine kinases such as everolimus (mTOR), crizotinib (ALK/HFGR), dasatinib (ABL/PTKSrc/EAR2), erlotinib (EGFR), lapatinib (EGFR/RTKErbB2), perifosine (Pi3K-AKT1), and midostaurin (FLT3/PDGFR/VEGFR/PKCa/c-Syk, c-Fgr/c-Kit and CDK1). Everolimus, crizotinib and erlotinib were tested in previous works and midostaurin has also been reported as a potent antiproliferative drug in high-throughput screening (HTS) in different SU-DIPG cells after 72 h of incubation, but no long-term efficacy data are available [8]. Meanwhile, lapatinib, perifosine, and dasatinib have never been tested in DIPG. In terms of PK properties dasatinib crosses the BBB and it is preferred over imatinib when CNS disease is present because imatinib has minimal BBB penetration. Everolimus has limited BBB permeation and is a substrate of the transporters encoded by the genes *ABCB1* and *ABCG2* being actively extruded but has been shown effective in some CNS diseases including DIPG. Lapatinib does cross the blood–brain barrier (BBB), but only to a limited extent, and its CNS exposure is restricted by efflux transporters as well as erlotinib. Crizotinib and perifosine do not effectively cross the BBB and neither does midostaurin. The antiproliferative effects of the unselective blocker of potassium channels, TEA, and the inwardly rectifying potassium (Kir) channel blocker, Ba^2+^ ion, were investigated as proof of concept.

## 2. Materials and Methods

### 2.1. Cell Culture

Patient-derived diffuse intrinsic pontine glioma (DIPG) cell cultures (SU-DIPG-36 and SU-DIPG-50) were supplied by Dr. Michelle Monje with approval from the Stanford University Institutional Review Board. These cells were cultured as a monolayer in tumor stem media (TSM), which is a 1:1 mixture of DMEM/F12 (Invitrogen, Carlsbad, CA, USA) and Neurobasal (-A) (Invitrogen), supplemented with B27 (-A) (Life Technologies, Carlsbad, CA, USA). The medium also included human basic fibroblast growth factor (bFGF) at 20 ng/mL, recombinant human epidermal growth factor (EGF) at 20 ng/mL, platelet-derived growth factor-AA (PDGF-AA) at 10 ng/mL, PDGF-BB at 10 ng/mL (Life Technologies), and heparin at 20 ng/mL (StemCell Technologies, Vancouver, BC, Canada). Cells were maintained at 37 °C in a 5% CO_2_ atmosphere.

### 2.2. Multi-Well Crystal Violet Staining Test

Cells were seeded on a 96-well plate at a density of 8 × 10^3^ cells. After 24 h, increasing concentrations of each drug were added. Following incubation periods of 48 and 72 h, the medium was removed, and cells were fixed with 10% buffered formalin for 20 min at 18–20 °C. They were then stained with 1% crystal violet (CV) for 30 min and analyzed at a wavelength of 560 nm. Each condition was performed at least four times. This procedure aims to quantify live, adherent cells in culture, as crystal violet binds to DNA and cell proteins [16].

### 2.3. CCK-8 Intracellular Dehydrogenase Assay

The activity of intracellular dehydrogenases was measured using the Cell Counting Kit-8 (CCK-8) (Sigma-Aldrich, St. Louis, MO, USA), which employs water-soluble tetrazolium salt [16]. Cells were counted and seeded into 96-well plates at a density of 8 × 10^3^ cells per well, then pre-incubated for 24 h under standard conditions. Subsequently, cells were treated for 48 and 72 h with different concentrations of each drug. The long-term response of the cells to MIDO 2.14 μM were investigated by monitoring the changes of Abs over time in the same 96-well plate. These experiments were performed in triplicate in SU-DIPG cells, in murine bone marrow cells [28] and in neuroblastoma SHSY5Y (ATCC CRL-2266) cells following differentiation with retinoic acid as previously described [29].

### 2.4. Clonogenic Assay

The clonogenic assay aimed to assess the cells’ ability to form colonies. Cells, seeded onto 60 mm plates in a population of 250 cells/plate, were counted using the Scepter 2.0 cell counter. In this assay, we examined how the substance affected colony formation after 72 h. Each condition was tested in triplicate. Following treatment, the culture medium containing the cytotoxic compound was replaced with fresh, drug-free medium, and cells were cultured for an additional two weeks. Colony analysis was performed using OpenCFU 3.9.0 (My Biosoftware, San Diego, CA, USA), supplemented by manual counts from three operators, with the average taken for results [30].

### 2.5. Impedentiometry Cell Count and Volume Assay

The cell size was measured and counts were performed using the Scepter™ 2.0 cell counter (MERK-Millipore, Billerica, MA, USA).

### 2.6. Protein Analysis by Western Blot

Western blotting was conducted using whole cell lysates, following the previously described method [16]. To identify apoptotic markers, Cell Signaling Technology reagents were employed. The detection of immunoreactive bands involved horseradish-peroxidase-labeled secondary antirabbit IgG and antimouse IgG (Cell Signaling Technology, Danvers, MA, USA), along with enhanced chemiluminescence (Pierce, Thermo Fisher Scientific, Waltham, MA, USA). Details are provided in Table S1 for mAb. Images and densitometry data were analyzed with Image Lab software 6.1 (Bio-Rad, Hercules, CA, USA).

### 2.7. Whole-Cell Recordings in the Cells

Ion channel currents in DIPG cells were measured using a whole-cell patch clamp by applying a depolarization protocol. The potential ranged from −100 to +180 mV (Vm) in 20 mV steps, with the holding potential (HP) set at −60 mV (Vm), and included asymmetrical K^+^ ion concentrations. Recordings were performed at room temperature (20–22 °C) with a sampling rate of 2 kHz (filtering at 1 kHz) using an Axopatch-1D amplifier paired with a CV-4 head-stage (Axon Instruments, Foster City, CA, USA). Patch pipettes were pulled from PG52165-4 #8250 glass capillaries, measuring 1.65 mm in outer diameter and 1.20 mm in inner diameter (World Precision Instruments, 175 Sarasota Center Blvd., Sarasota, FL, USA), using a vertical puller (PP-82 Narishige, Tokyo, Japan) to achieve a resistance of 4–5 MΩ. Cells that did not form a seal with resistance greater than 1 GΩ were not considered for the analysis.

### 2.8. Drugs and Solution

Ion channel activators and inhibitors such as glibenclamide (GLIB), repaglinide (REPA), tetraethylammonium chloride (TEA), Ba^2+^ ions, ruthenium red (RR), and capsazepine (CAPSZ) were sourced from Sigma (Sigma-Aldrich, Merck KGaA, Darmstadt, Germany). Stock solutions of GLIB, REPA, TEA, BaCl_2_, and CAPSZ were prepared by dissolving each compound in dimethyl sulfoxide (DMSO) at concentrations of 118.6 mM; RR was dissolved in water. During experiments, tested substances included everolimus (EVE), dasatinib (DASA), perifosine (PERIF), crizotinib (CRIZO), lapatinib (LAPA), erlotinib (ERLO), and midostaurin (MIDO). Their stock solutions were made by dissolving each drug in DMSO and stored at −20 °C. Dilutions with Dulbecco’s modified Eagle medium (DMEM) provided solutions at required concentrations, prepared on the experimental day and kept at room temperature.

For patch-clamp experiments, small volumes of these stock solutions were added to the bath solutions provided by different protocols. DMSO concentration did not exceed 0.07%, which typically does not influence ion channel current or cell proliferation. The bath solution comprised 142 mM NaCl, 2.8 mM KCl, 1 mM CaCl_2_, 1 mM MgCl_2_, 11 mM glucose, and 10 mM HEPES (pH 7.4). The pipette solution included 132 mM K^+^-glutamate, 1 mM EGTA, 10 mM NaCl, 2 mM MgCl_2_, 10 mM HEPES, 1 mM Na_2_ATP, and 0.3 mM Na_2_GDP (pH 7.2).

### 2.9. Data Analysis and Statistics

Statistical results are shown as mean ± SEM unless otherwise specified. Data collection and analysis were conducted using Excel (Microsoft Office 2010) (Microsoft Corporation, Redmond, WA, USA) and GraphPad Prism 8 to calculate IC_50_, Emax, and Hill slope through curve fitting algorithms. Significant differences between two group means were determined with *p* values < 0.05 using Student’s *t*-test. Additionally, one-way ANOVA was employed to compare the means of three or more groups, assessing whether at least one differs significantly. This test was used to examine differences across independent groups and within groups to analyze variations in means within the same group. Patch-clamp data acquisition and analysis were carried out using the pCLAMP 10 software suite (Axon Instruments, Foster City, CA, USA).

SCOPUS search database key words: drug name AND DIPG AND “diffuse intrinsic pontine glioma” AND “Diffuse midline glioma” AND DMG.

## 3. Results

### 3.1. Effects of Tyrosine Kinase Inhibitors in “In Vitro” Cell Viability Experiments on SU-DIPG-36

The antiproliferative effects of different TKIs were investigated in neuronal SU-DIPG-36 in CV and CCK-8 assays. A concentration selecting protocol for each drug was performed. We firstly tested two concentrations of each drug effective against their targets reported in the literature to evaluate efficacy, the data were reported in box-plot analysis and efficacy confirmed in a clonogenic assay. The 100 μM concentration for some drugs such as everolimus and perifosine, despite not being clinically relevant, was tested because some living cells were still observed in the plates when testing these drugs at nanomolar concentrations, particularly with perifosine. We then perform full concentration–response experiments (0.001–100 μM). Crystal violet labels nuclear DNA in adherent cells while the CCK-8 assay is a functional assay and the cell count is based on evaluation of mitochondrial dehydrogenase activity. The crystal violet (CV) assay and the CCK-8 assay measure different parameters of cell viability, which is why they can give divergent results. CV quantifies adherent cells but does not provide information on their functional status. CCK-8, instead, detects metabolic activity through the reduction of WST-8 by intracellular dehydrogenases: the signal may decrease due to metabolic inhibition even in the absence of cell loss or be relatively high if few cells remain metabolically active. This can explain why in some experiments we obtain different results for the same drugs tested with different assays.

**Figure 1:**
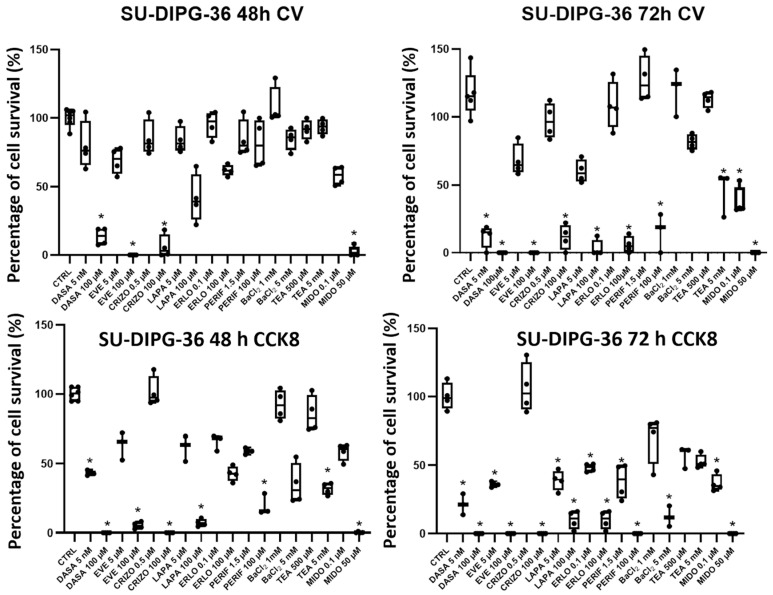
Box-plot analysis of cell survival percentage (%) changes with TKI in SU-DIPG-36 cells after 48 h and 72 h of incubation in 96-well crystal violet (CV) and CCK-8 assays. Dasatinib (DASA) (5 nM and 100 µM), everolimus (EVE) (5 µM and 100 µM), crizotinib (CRIZO) (0.5 µM and 100 µM), lapatinib (LAPA) (5 µM and 100 µM), erlotinib (ERLO) (0.1 µM and 100 µM), perifosine (PERIF) (1.5 µM and 100 µM), BaCl_2_ (1 mM and 5 mM), TEA (500 µM and 5 mM) and midostaurin (MIDO) (0.1 µM and 50 µM). One-way ANOVA test showed data significantly different within and between groups with F values > 2 (*). At least three samples per condition were selected for data representation. The data are the mean ± SEM. Data are normalized to CTRL data, data exceeding 100% survival can be observed in some cases when the cell number is higher than CTRL.

DASA 100 μM, EVE 100 μM, CRIZO 100 μM and 50 μM MIDO were markedly effective in the CV assay after 48 h of incubation time, decreasing cell survival. After 72 h of incubation time the effects were also observed with PERIF 100 μM, LAPA 100 μM, ERLO 100 μM and with low concentrations of DASA 5 nM and MIDO 0.1 μM (Figure 1). In the CCK-8 assay, the effects of the TKIs were also markedly observed (Figure 1). The unselective blocker of potassium channels, TEA, and the inwardly rectifying potassium (Kir) channels blocker, Ba^2+^ ion, concentration-dependently reduced cell proliferation in CCK-8 assays. TEA also blocks calcium-activated K^+^ channels (BK) and aquaporin channels.

In some cases, we can see an increase in cell viability between 48 h and 72 h of incubation in experiments where cells are treated with low drug concentrations and prolonged incubation time, such as for PERIF (1.5 µM), ERLO (0.1 µM) and BaCl_2_ (1 mM). This may be because low concentrations of drugs may not be sufficient to inhibit growth but, on the contrary, may induce an adaptive response that stimulates survival over time.

### 3.2. Clonogenic Assay on SU-DIPG-36 Performed to Investigate Substances Proving Greater Efficacy

**Figure 2:**
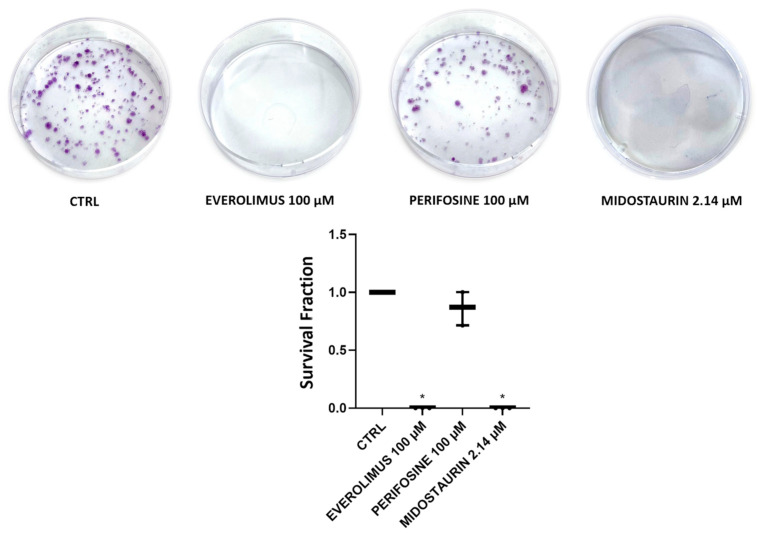
Clone formation with everolimus 100 µM (EVE) and perifosine 100 µM (PERIF) and 2.15 μM midostaurin (MIDO) on SU-DIPG-36 using the clonogenic assay after 72 h of incubation. It should be noted in this assay that the drug solution is washed out following treatment so that survival cells, if any, can form a colony. Student *t*-test for *p* < 0.05 * was used to test for significance between the two groups. At least three samples per condition were selected for data representation. The data are the mean ± SEM.

**Figure 3:**
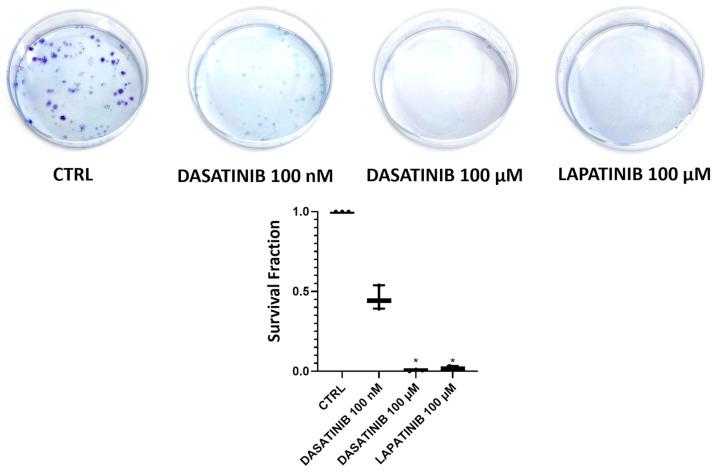
Clone formation with dasatinib (DASA) 100 nM and 100 µM and lapatinib (LAPA) 100 µM on SU-DIPG-36 using the clonogenic assay after 72 h of incubation. It should be noted in this assay that the drug solution is washed out following treatment, so that survival cells, if any, can form a colony. Student *t*-test for *p* < 0.05 * was used to test for significance between the two groups. At least three samples per condition were selected for data representation. The data are the mean ± SEM.

In the clonogenic assay on SU-DIPG-36 cells, 2.14 μM MIDO and 100 μM EVE fully reduced the number of colonies after 72 h of incubation time following washout of the drug solution but not 100 μM PERIF (Figure 2; Table 1). LAPA and DASA at 100 μM concentrations were fully effective in this assay, as well as DASA at a nanomolar concentration (Figure 3; Table 2).

Midostaurin, which was already proven to be effective at low concentrations in crystal violet and CCK-8 assays, eliminated colony formation at 2.14 µM concentrations, with no detectable colonies in the clonogenic assay. This result shows that the minimum concentration needed to achieve the maximum effect is ≤2.14 µM in the cell line tested. Instead, with the other substances tested, higher concentrations are needed to achieve the maximum effect, which is why the 100 μM concentration had to be tested for the other drugs in the clonogenic assay.

### 3.3. TKI-Induced Concentration-Dependent Inhibition of Cell Proliferation in SU-DIPG Cells

**Figure 4:**
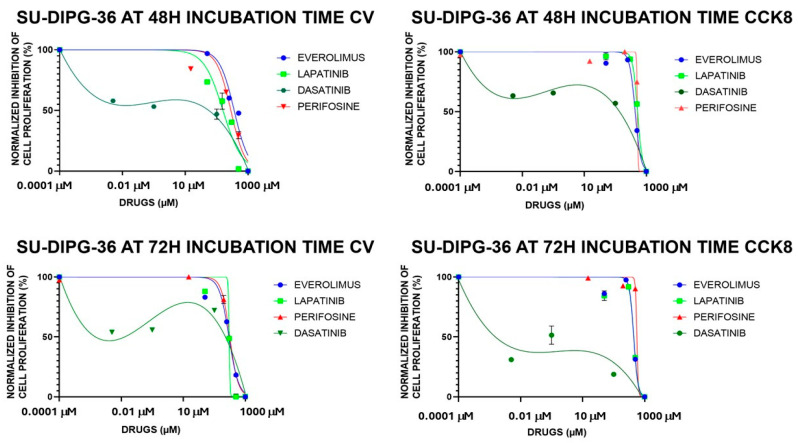
Everolimus (EVE), lapatinib (LAPA), perifosine (PERIF), dasatinib (DASA) and midostaurin (MIDO) (0.001–100 μM) concentration-dependent inhibition of cell proliferation, expressed as normalized percentage (%), on SU-DIPG-36 cells in multi-well crystal violet (CV) and CCK-8 assays after 48 h and 72 h of incubation. The data points are the means ± S.D. of at least three replications for each concentration.

A concentration-dependent reduction of cell proliferation was seen with EVE, LAPA, PERIF, CRIZO, and ERLO (0.01–100 μM) in the micromolar ranges. But MIDO and DASA (0.001–100 μM) at all investigated incubation times reduced cell viability at lower concentrations (Figure 4). MIDO was the most potent TKI in SU-DIPG-36 cells, showing a monophasic concentration–response relationship vs. the other drugs. Meanwhile, the concentration-dependent response of DASA was biphasic with lower efficacy at higher concentrations (Figure 4; Table 3).

The biphasic concentration–response relationship of DASA suggests that dasatinib may affect multiple targets with different affinities. At very low concentrations, inhibition of a high-affinity target induces strong cytotoxicity. At intermediate concentrations, compensatory or adaptive pathways may be activated that partially saves cells viability, reducing clear inhibition. At high concentrations, simultaneous inhibition of multiple kinases or non-specific effects restore complete suppression of proliferation, creating what we consider a biphasic effect.

**Figure 5:**
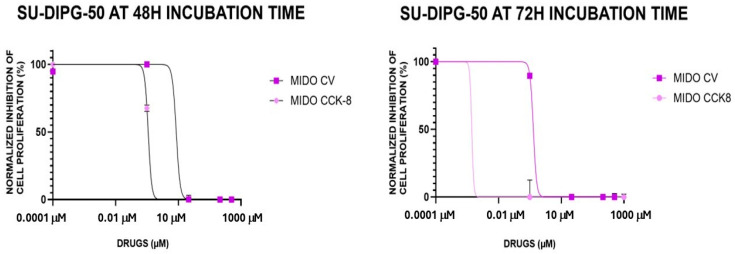
Midostaurin (MIDO) (0.001–100 μM) concentration-dependent inhibition of cell proliferation, expressed as normalized percentage (%), on SU-DIPG-50 cells in multi-well crystal violet (CV) and CCK-8 assays after 48 h and 72 h of incubation. The data points are the means ± S.D. of at least three replications for each concentration.

MIDO was therefore investigated in SU-DIPG-50; we found that it caused a −80.8 ± 8% and −100% change in cell proliferation after 48 h and 72 h of incubation, respectively. Concentration–response curve fitting analysis revealed that the IC_50_ (M) values to reduce cell proliferation were in sub-micromolar concentration ranges in all experimental conditions and in a nanomolar concentration range in the CCK-8 assay (Figure 5; Table 4) after 72 h.

### 3.4. Mechanism of Action of MIDO in SU-DIPG Cells: Characterization of Ion Channel Currents in SU-DIPG Cells

**Figure 6:**
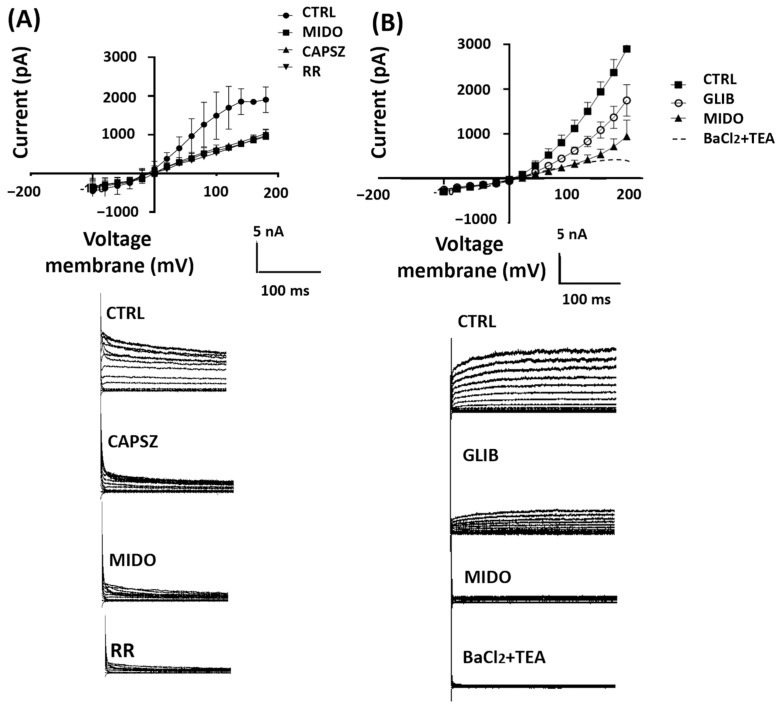
I/V relationships and effects of midostaurin (MIDO) on the macroscopic K^+^ currents recorded in SU-DIPG-36 cells during voltage step application to the cells. (**A**) Sample traces of CTRL current, after the application of CAPSZ, MIDO and RR. The application of 1 μM CAPSZ, 2.14 μM MIDO, and 10 μM RR reduced control currents with 10 μM RR being the most effective compound. (**B**) Sample traces of CTRL current, after the application of GLIB, MIDO and RR. The application of GLIB 100 μM, 2.14 μM MIDO and 5 mM BaCl_2_ + 10 mM TEA reduced control currents with 5 mM BaCl_2_ + 10 mM TEA being the most effective drugs. A washout period of 5 sec follows the application of drug solutions.

Whole cells’ currents with a hyperbolic current–voltage relationship can be recorded in both cell types in physiological K^+^ ion concentrations. The SU-DIPG-36 cells (N cells = 30) had a resting potential of −11.2 ± 12 mV (Vm) and capacitance of 4.02 ± 7 pF. Patch-clamp investigations showed that the TRPV1 antagonist 10 μM CAPSZ reduced the control currents (Figure 6A) (N cells= 10) by −60% ± 9 at +40 mV (Vm) and −49% ± 9 at +100 mV (Vm). The addition of MIDO reduced the CTRL currents that were CAPSZ-sensitive by −48% ± 10 at +80 Vm (Vm) in the cells (Figure 6A). The CTRL currents were further reduced by the unselective TRP antagonist 10 μM RR. GLIB at 100 μM also reduced the CTRL currents in these cells (N cells = 20) that were further reduced by MIDO (Figure 6B); these currents were fully reduced by TEA and BaCl_2_ that are not selective K^+^ channel blockers (Figure 6B).

These findings suggest that the observed reduction of CTRL current by MIDO is due to inhibition of TRPV1 and KATP channels in the SU-DIPG-36 cells.

Although we are dealing with the same cell type in these experiments, we can see that the control current trend between +150 and +200 mV is slightly different, which may depend on intrinsic cell variability, as the trend stays the same in the rest of the range.

**Figure 7:**
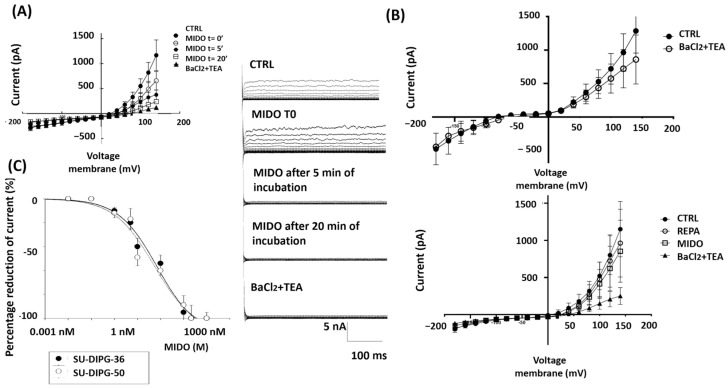
I/V relationships and effects of midostaurin (MIDO) on the macroscopic K^+^ currents recorded in SU-DIPG-50 cells during voltage step application to the cells. (**A**) Sample traces of CTRL current, after the application of MIDO at t = 0 and after 5 and 20 min of incubation. The application of 2.14 μM MIDO at different incubation times reduced the currents time-dependently. (**B**) I/V relationships of cells with resting potentials close to the equilibrium potentials for K^+^ ions (upper panel) and of markedly depolarized cells sensitive to 100 μM REPA, a KATP channel inhibitor, and 2.14 μM MIDO (lower panel). (**C**) Concentration–response relationships of MIDO concentrations vs. ion channel currents at −60 mV (Vm) in SU-DIPG-36 and SU-DIPG-50 cells. The data points are the mean ± SEM of at least three replications for each drug concentration.

In some SU-DIPG-50 cells (N cells = 3) with resting potential of +20 ± 9 mV (Vm) and capacitance of 8 ± 11 pF, 2.14 μM MIDO reduced currents vs. controls at positive membrane potential after 20 min of incubation time, but at t= 0 it failed to inhibit this current at negative potentials. An increasing inhibitory effect was seen over time in these same cells at positive potentials (Figure 7A, Table 5). SU-DIPG-50 cells were therefore markedly depolarized with I/V crossing the voltage axis at +23 ± 8 mV (Vm) and a membrane capacitance of 3.02 ± 10 pF (N cells = 4) and were sensitive to REPA, the KATP channel inhibitor, and MIDO (Figure 7B, Table 6).

We also found some cells (N cells = 3) with hyperbolic current–voltage relationships crossing the I/V at −90 ± 8 mV (Vm) (membrane capacitance of 3.01 ± 8 pF) close to the equilibrium potential for K^+^ ions like normal neurons that were sensitive to 5 mM BaCl_2_ and 10 mM TEA (Figure 7B, Table 6).

The fact that BaCl_2_ + TEA shows a slightly different trend among the panels presented is related to the treatments carried out before BaCl_2_ + TEA, which certainly change the electrical state of the cell.

Concentration–response analysis calculated an IC_50_ of MIDO of 71.3 nM (Hill slope = −0.5) on SU-DIPG-36 cell current and of 69 nM (Hill slope = −0.45) on SU-DIPG-50 cell current (Figure 7C).

### 3.5. Long-Term Response of SU-DIPG-36 and SU-DIPG-50 Cells to MIDO and Effect on Murine Bone Marrow Cells and SHSY5Y Cells

**Figure 8:**
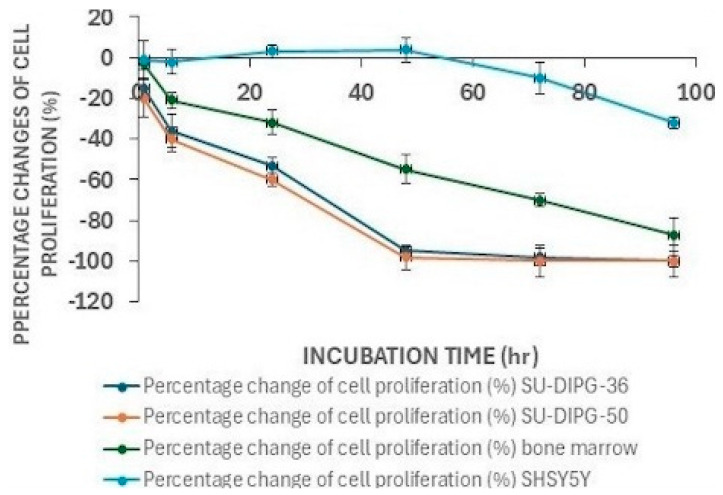
The long-term response of the cells was evaluated monitoring the Abs changes in the same 96-well plates at different incubation times. The data points are the mean ± SEM of at least three replications.

The long-term responses of SU-DIPG cells to MIDO were evaluated. The incubation of the cells with 2.14 μM MIDO caused a comparable time-dependent reduction of cell proliferation in SU-DIPG-50 and SU-DIPG-36 cells vs. controls, with significant reduction after 6 h of incubation. Murine bone marrow cells were less responsive to MIDO in the short term, but a marked reduction of cell proliferation was seen and comparable to that in the SU-DIPG cells after 96 h of incubation. In contrast, differentiated SHSY5Y cells were not responsive to MIDO after short-term incubation and much less responsive after 96 h of incubation (Figure 8).

### 3.6. Effects of MIDO on Target Proteins After 48 h of Incubation Time in SU-DIPG Cells

Due to the observed efficacy of MIDO particularly in the SU-DIPG-36 cells after 48 h of incubation, we investigated some MIDO targets such as VEGFR2 and PDGFR in both cell lines by Western blotting using β-actin as a normalization standard. We also investigated the effects of MIDO on DIPG targets such as the mTOR/AKT and H3K27ac contents that were firstly evaluated and are expected to be elevated following treatment with antiproliferative HDAC drugs in DIPG [31].

**Figure 9:**
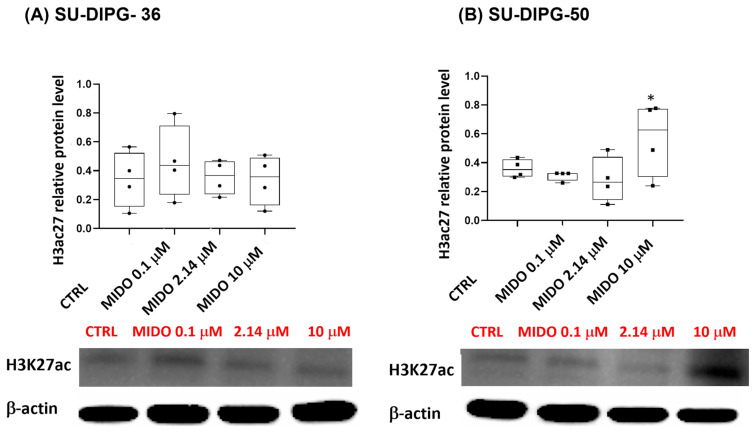
Effects of MIDO on the expression of DIPG targets. Acetyl histone H3 protein expression in SU-DIPG-36 and SU-DIPG-50 cells after treatment with increasing concentrations of MIDO after 48 h of treatment. (**A**) Box-plot representations of acetyl histone H3 (Lys27) content in SU-DIPG-36 cells. Western blotting images show acetyl histone H3 content, below gel panel. (**B**) Box-plot representations of acetyl histone H3 (Lys27) content in SU-DIPG-50 cells. Western blotting images show acetyl histone H3 content, below gel panel. β-actin was used as a housekeeping protein. The actin panels were obtained from the same gels and captured under the same exposure conditions as the target proteins. All data were normalized to β-actin. Data significantly different by ANOVA * *p* < 0.05 vs. the control group (CTRL). Box-plot data are the means ± SEM of three replications.

MIDO did not affect H3K27ac protein content at all concentrations in SU-DIPG-36 and markedly increased it only at a 10 μM concentration in SU-DIPG-50 (Figure 9A,B; Figures S1, S2, S5–S7, S12 and S14–S16). The disease is monoallelic with possible enhancement of the normal H3K27ac in our experiments following MIDO in SU-DIPG-50. The antibody used in Western blot experiments (Table S1) does not distinguish between the two forms, so we believed that we saw the upregulation of the normal allele. In our earlier work, the KATP channel inhibitor repaglinide concentration-dependently enhanced H3K27 acetylation in both SU-DIPG cells with antiproliferative effects in the sub-nanomolar concentration range using the same mAbs for Western blot experiments [16].

**Figure 10:**
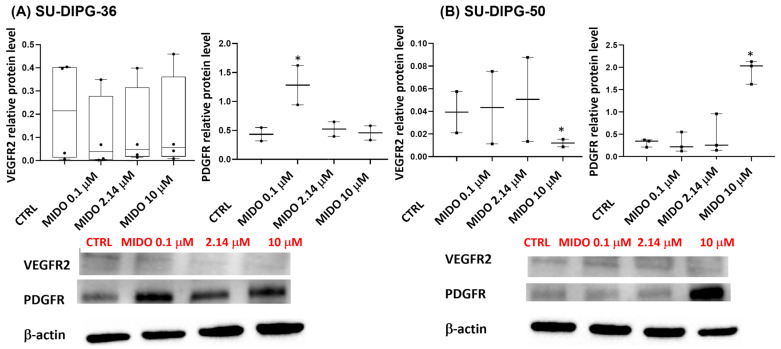
Effects of increasing concentrations of MIDO on the expression of its targets VEGFR2 and PDGFR in SU-DIPG-36 and SU-DIPG-50 cells after 48 h of incubation. (**A**) Box-plot representations of VEGFR2 and PDGFR content in SU-DIPG-36 cells. Western blotting images showing VEGFR2 and PDGFR protein expression, below gel panel. (**B**) Box-plot representations of VEGFR2 and PDGFR content in SU-DIPG-50 cells. Western blotting images showing VEGFR2 and PDGFR protein content, below panel. β-actin was used as a housekeeping protein. The actin panels were obtained from the same gels and captured under the same exposure conditions as the target proteins. All data were normalized to β-actin. Data significantly different by ANOVA * *p* < 0.05 vs. the control group (CTRL). Box-plot data are the mean ± SEM of three replications.

MIDO decreased the target receptor VEGFR2 level in SU-DIPG-50 at a high 10 μM concentration with no effects on SU-DIPG-36 (Figure 10A,B, Figures S1, S2, S5 and S11). This drug paradoxically upregulated PDGFR in both cells (Figure 10A,B, Figures S12 and S13).

MIDO affected mTOR/AKT content, but not significantly, in SU-DIPG-36 cells (Figure 11A, Figures S3, S4, S10 and S17). However, MIDO at a sub-micromolar concentration significantly downregulated mTOR in the SU-DIPG-50 cells. A high concentration of 10 μM caused mTOR dephosphorylation in SU-DIPG-50 (Figure 11B, Figures S6–S9 and S17).

Phospho-ERK1-2 and ERK1-2 contents were not changed following 48 h of treatment of both cells with MIDO (Figures S11–S13).

**Figure 12:**
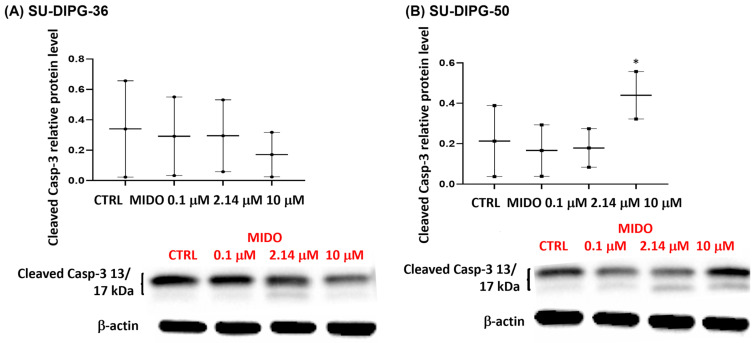
Expression of cleaved caspase-3 in SU-DIPG-36 and SU-DIPG-50 cells after treatment with increasing concentrations of MIDO after 48 h of incubation. (**A**) Box-plot representations of cleaved caspase-3 content in SU-DIPG-36 cells. Western blotting images showing cleaved caspase-3 protein content, below gel panel. (**B**) Box-plot representations of cleaved caspase-3 content in SU-DIPG-50. Western blotting images showing cleaved caspase-3 protein content, below gel panel. β-actin was used as a housekeeping protein. The actin panels were obtained from the same gels and captured under the same exposure conditions as the target proteins. All data were normalized to β-actin. Data significantly different by ANOVA * *p* < 0.05 vs. the control group (CTRL). Box-plot data are the mean ± SEM of three replications.

A concentration-dependent decrease in the apoptotic marker cleaved caspase-3, following 48 h of treatment of SU-DIPG-36, was seen with MIDO (Figure 12A). But SU-DIPG-50 cells treated with 10 μM MIDO showed an enhanced content of this protein (Figure 12B). This finding supports the idea that caspase-3 is not a target of MIDO action in SU-DIPG-50 cells.

**Figure 13:**
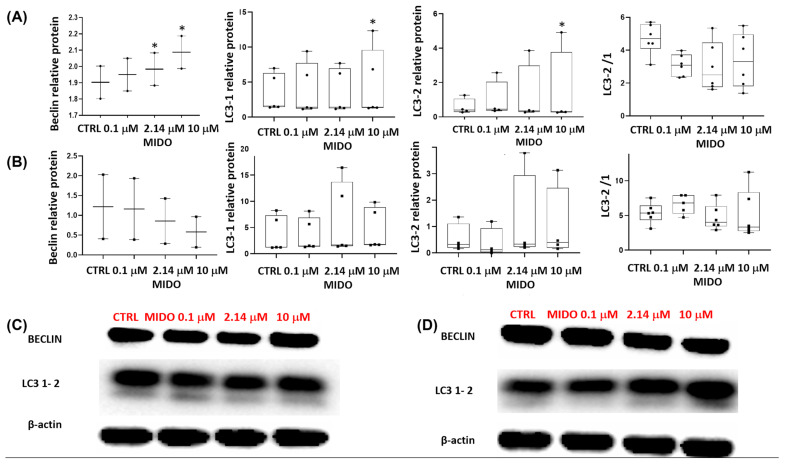
Expression of Beclin and LC3-1/2 in SU-DIPG-36 and SU-DIPG-50 cells after treatment with increasing concentrations of MIDO after 48 h of incubation. (**A**) Box-plot representations of Beclin and LC3-1/2 content in SU-DIPG-36 cells. (**B**) Box-plot representations of Beclin and LC3-1/2 content after 48 h of treatment in SU-DIPG-50 cells. (**C**) Western blotting images showing Beclin and LC3-1/2 protein content in SU-DIPG-36 cells. (**D**) Western blotting images showing Beclin and LC3-1/2 protein content in SU-DIPG-50. β-actin was used as a housekeeping protein. The actin panels were obtained from the same gels and captured under the same exposure conditions as the target proteins. All data were normalized to β-actin. Data significantly different by ANOVA * *p* < 0.05 vs. the control group (CTRL). Box-plot data are the mean ± SEM of three replications.

We also investigated some autophagic markers such as Beclin and LC3-1–2 and found a concentration-dependent increase in Beclin and LC3-1 and 2 content in the SU-DIPG-36 cells (Figure 13A,C, Figures S9 and S10) while a concentration-dependent decrease in Beclin was observed in the SU-DIPG-50 cells (Figure 13B,D). The LC3-2 and LC3-1 contents were not affected by MIDO in SU-DIPG-50 and Beclin was downregulated, supporting the idea of MIDO-induced autophagy in the SU-DIPG-36 cells (Figures S9 and S10).

## 4. Discussion

Tyrosine kinase inhibitors (TKIs) that are approved for different cancers are under investigation in brain tumors [32,33,34]. However, there is to date no tyrosine kinase inhibitor approved for brain tumor treatments such as glioblastoma or DIPG [35,36].

We firstly compared the effects of TKIs targeting different kinases such as everolimus (mTOR), crizotinib (ALK/HFGR), dasatinib (ABL/PTKSrc/EAR2), erlotinib (EGFR), lapatinib (EGFR/RTKErbB2), perifosine (Pi3K-AKT1) and midostaurin (FLT3/PDGFR/VEGFR/PKC/c-Syk, c-Fgr/c-Kit and CDK1) in SU-DIPG-36 cells. In box-plot analysis, everolimus, lapatinib, midostaurin, dasatinib, and perifosine were effective at low concentrations, at different incubation times and in CCK-8 and CV assays and were investigated for their concentration–response relationships.

Concentration–response relationship analysis revealed that all drugs have IC_50_ values in the micromolar concentration range except for midostaurin and dasatinib that showed IC_50_ values in sub-micromolar concentration ranges. Dasatinib showed, however, a bimodal concentration–response relationship, reducing its efficacy at high concentrations. This may be due to signaling escaping the inhibitory effects of dasatinib.

Midostaurin was the most potent and effective drug and therefore investigated in SU-DIPG-50 cells. In box-plot analysis, midostaurin confirmed its efficacy in SU-DIPG-50 cells at different incubation times and in either CV or CCK-8 assays. Concentration–response relationship investigations revealed that midostaurin has IC_50_ values in the sub-micromolar concentration range in all conditions and in the nanomolar concentration range after 72 h of incubation time in the CCK-8 assay.

The actions of the drug were investigated on the ion channel currents by patch clamp and on the phosphorylation of target proteins in DIPG by Western blotting. Considering that staurosporine, a structural analog and precursor of MIDO, is also known to reduce chloride and ATP-dependent potassium channel activity in other tissues [22,23], we tested the ability of MIDO to reduce ion channel current through patch-clamp experiments. Patch-clamp investigations show that the acute application of 2.14 μM midostaurin reduced the whole-cell inward and outward cation currents vs. controls in the presence of low internal ATP. These currents were sensitive to the KATP channel inhibitors glibenclamide and repaglinide and were reduced by the unselective blockers TEA and BaCl_2_. Midostaurin also reduced the capsazepine-sensitive currents, and these currents were further reduced by ruthenium red, supporting the idea that MIDO also reduced the TRPV1-sensitive currents. In earlier RT-PCR experiments, the expression of the *ABCC8* gene encoding for the SUR1 subunit and *TRPV1* genes in SU-DIPG-50 was higher than that observed in SU-DIPG-36, suggesting that these subunits can mediate MIDO actions in SU-DIPG-50 cells [16]. In SU-DIPG-50 the effects of MIDO were also time-dependent, leading to a full reduction of the control current after 20 min of incubation, suggesting an additional mechanism based on the dephosphorylation of ion channel subunits. Several ion channels have tyrosine consensus sites for phosphorylation by kinases that regulate channel openings. The action of midostaurin can therefore be direct as previously seen in excised membrane patches with staurosporine [23] and/or indirectly mediated by tyrosine kinases. One possible candidate is PKC. This enzyme is known to target the Kir6.1/2 C-terminal cytoplasmic regions and/or SUR1/2 intracellular loops, reducing the ATP sensitivity of these regions with channel opening [37,38]. PKC phosphorylation shifts the IC_50_ of ATP by stabilizing the open state of the channel, reducing ATP’s ability to close it [38]; kinase inhibitors like staurosporine and midostaurin can reverse this effect by blocking PKC signaling. PKC also modulates TRPV1 by phosphorylating intracellular regulatory residues, which lowers activation thresholds, stabilizes the open state, and suppresses desensitization—sensitizing the channel without directly opening it or altering ligand binding [26,27]; this is why inhibitors of PKC reduce TRPV1 channel currents as observed in our experiments. The hyperbolic I–V relationships that we observed in our experiments may reflect partial modulation rather than direct channel blocking. Some pharmacological agents, particularly kinase inhibitors or membrane-active compounds, can alter channel gating kinetics or voltage dependence rather than producing a classical pore block. Such modulation can manifest as a change in the curvature of the I–V relationship [38,39].

The fact that the calculated IC_50_ values of MIDO on the ion channel currents in either SU-DIPG cells were in the range of the IC_50_ values of antiproliferative drugs supports the action of MIDO as an ion channel inhibitor. But the involvement of other ion channels cannot be excluded to date.

Here, we further saw that the unselective blockers of Kir and Kv channels, respectively, BaCl_2_ and TEA used here as a tool, are also effective in inhibiting the ion channel currents and antiproliferative compounds, supporting the role of K^+^ ion channels in regulating cell proliferation in these cells but with different efficacy. Ba^2+^ ions were indeed more effective than TEA in our experiments after 72 h of incubation in the mitochondrial dehydrogenase activity assay. As a pore blocker of Kir6.1/2 subunits of KATP channels, Ba^2+^ ions show a mild antiproliferative response, while the sulfonylurea drugs binding to SUR1/2 subunits are very effective, supporting the idea that these subunits play a major role as antiproliferative targets in SU-DIPG cells [16]. Relevant studies have identified and characterized potassium channels’ involvement in glioblastoma multiforme (GBM) progression and plasticity in concert with TRP channels. In this aggressive form of brain cancer the tumor cells show an abnormally elevated calcium-activated K^+^ channel activity also mediated by the BK channel associated with elevated expression of the chloride channels VRAC and TRPM [40,41]. These ion channel activities promote hypotonic volume regulation with migration and invasiveness in glioblastoma multiforme. 

In SU-DIPG-36 cells MIDO caused a concentration-dependent upregulation of autophagy markers and a decrease in cleaved caspase-3 in the effective antiproliferative range of concentrations, suggesting a major contribution of autophagic cell death in this cell line. In these cells we also found the induction of PDGFR which has been recently associated with autophagic regulation in cancer [42,43,44]. Some PKC isoforms indirectly regulate Beclin-1 and LC3 activity through upstream pathways, including mTOR signaling that inhibits autophagy, so that PKC inhibitors like midostaurin can potentiate autophagy. However, we found that ERK1/2 which is a pathway activated by survival molecules like FGF23 and klotho and a drug target in cancers is not involved in midostaurin actions in SU-DIPG cells.

In SU-DIPG-50 cells, MIDO at a high concentration of 10 μM causes a downregulation of VEGFR2, the enhancement of the acetyl histone H3 content and cleaved caspase-3, and it is therefore unlikely that these targets would contribute to antiproliferative effects in vivo.

Therefore, the downregulation of mTOR in SU-DIPG-50 and the activation of the autophagic pathway in SU-DIPG-36 cells combined with the inhibitory actions of ion channels observed in both cell lines possibly mediated by the PKC dephosphorylation of the target proteins explains the antiproliferative action of MIDO in these cells.

The link between potassium channels as well as TRP channels and autophagy is well described since alteration of potassium and calcium homeostasis can regulate autophagy through upregulation of Beclin and LC3-2 as observed in our experiments [45].

Bibliographic analysis found no experimental data for lapatinib, perifosine, and dasatinib in DIPG, while promising experimental data for erlotinib, midostaurin and everolimus were reported [8]. In our experimental setting of SU-DIPG-50 vs. SU-DIPG-36 cells, MIDO was more potent than everolimus and erlotinib with stable response all over 72 h of incubation. Long-term antiproliferative responses were seen with saturating test concentrations of 2.14 μM midostaurin in both cell lines after 96 h of incubation, being 10-fold less effective in bone marrow cells and not effective in neuroblastoma cells. MIDO shows favorable safety factors with antiproliferative IC_50_ values in the sub-micromolar concentration range that are in the range of the reported Cmax of the free drug concentration of 3.5–35 nM in a steady state calculated for 50 mg per os twice daily [46,47]. Midostaurin 0.1 μM is also considered a weak inhibitor of hERG with IC_50_ values >10 μM (Rydapt^®^) and low cardiovascular risk.

It should be noted that midostaurin, which is a semi-synthetic derivative of staurosporine [46,47] that affects hundreds of kinases, has never been reported to be effective in brain cancers, having a low BBB permeability [48]. Despite this, in one paper, midostaurin shows a CNS-protective effect following intraperitoneal injection (25 mg/kg) in C6/7 injured Wistar rats [45]. The low BBB permeability of chemotherapeutic drugs is a common issue in DIPG, and several groups are working on this using innovative approaches [49,50,51].

## 5. Conclusions

The inhibition of cation channel currents by midostaurin observed in SU-DIPG-36 and SU-DIPG-50 cells and the autophagy potentiation in SU-DIPG-36 cells can be a novel mechanism in DIPG. The emerging role of ion channels in cancers and the developments of new data obtained in high-throughput screening (HTS) and planar patch clamp studies support the idea that cancer is a channelopathy [52].

It should be noted that inhibition of different types of K^+^ channels in heart cells, including KATP channels, can handle arrhythmias, while inhibition of TRPV1 is associated with loss of analgesic response [53].

## Figures and Tables

**Figure 11:**
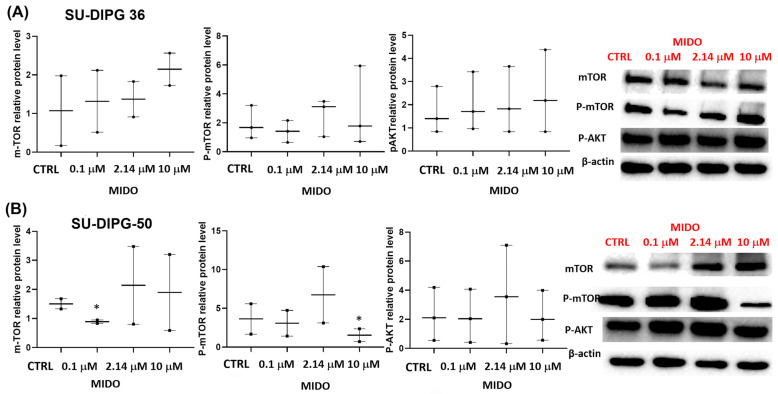
Effects of MIDO on the expression of DIPG targets. Expression of mTOR and AKT in SU-DIPG-36 and SU-DIPG-50 cells after treatment with increasing concentrations of MIDO after 48 h of incubation. (**A**) Box-plot representations of mTOR, phospho-mTOR and phospho-AKT contents and sample Western blotting images showing mTOR, phospho-TOR, and phospho-AKT protein content. β-actin was used as a housekeeping protein. (**B**) Box-plot representations of mTOR, phospho-mTOR and phospho-AKT in SU-DIPG-50 and sample Western blotting images showing these protein contents. β-actin was used as a housekeeping protein. The actin panels were obtained from the same gels and captured under the same exposure conditions as the target proteins. All data were normalized to β-actin. Data significantly different by ANOVA * *p* < 0.05 vs. the control group (CTRL). Box-plot data are the mean ± SEM of three replications.

**Table 1:** Survival fraction (%) and *n* of formed colonies after 72 h of incubation for SU-DIPG-36 treated with everolimus 100 µM, perifosine 100 µM and midostaurin 2.14 µM.

Experimental Condition	Survival Fraction (%) After 72 h of Incubation	*n* of Formed Colonies After 72 h of Incubation
CTRL	100	186.3
Everolimus 100 µM	0.06 ± 0.0006	0.1
Perifosine 100 µM	86.2 ± 0.08	160.6
Midostaurin 2.14 µM	0 ± 0	0

**Table 2:** Survival fraction (%) and *n* of formed colonies after 72 h of incubation for SU-DIPG-36 treated with dasatinib 100 nM, dasatinib 100 µM and lapatinib 100 µM.

Experimental Condition	Survival Fraction (%) After 72 h of Incubation	*n* of Formed Colonies After 72 h
CTRL	100	89.2
Dasatinib 100 nM	45.7 ± 0.05	40.8
Dasatinib 100 µM	0.6 ± 0.003	0.5
Lapatinib 100 µM	2.05 ± 0.003	1.8

**Table 3:** Fitting parameters of Tyrosine Kinase Inhibitor (TKI) concentration–response relationships in SU-DIPG-36 cells in 96-well Crystal violet (CV) and CCK-8 assays after 48 h and 72 h incubation.

SU-DIPG-36 Cells	EVEROLIMUS IC_50_ (μM) Hill Slope Emax (%)	LAPATINIB IC_50_ (μM) Hill Slope Emax (%)	PERIFOSINE IC_50_ (μM) Hill Slope Emax (%)	DASATINIB IC_50_ (μM) Hill Slope Emax (%)	MIDOSTAURIN IC_50_ (μM) Hill Slope Emax (%)
CV 48 h	36.07 μM	15.87 μM	28.29 μM	0.1912 μM	0.2170 μM
	−1.870	−1.426	−1.937	−0.2534	−1.762
	−100%	−53.61%	−99.88%	−81.81%	−100%
CCK-8 48 h	43.61 μM	52.10 μM	51.59 μM	10.57 μM	0.9486 μM
	−4.869	−5.676	−3.475	−5.059	−2.064
	−91.80%	−92.77%	−65.47%	−100%	−100%
CV 72 h	29.54 μM	29.95 μM	31.54 μM	10.95 μM	0.3356 μM
	−2.805	−29.17	−3.246	−10.44	−1.311
	−100%	−100%	−100%	−100%	−100%
CCK-8 72 h	44.35 μM	44.47 μM	56.15 μM	14.78 nM	1.4739 μM
	−6.532	−6.161	−19.10	−0.294	−2.189
	−100%	−100%	−100%	−100%	−100%

**Table 4:** Fitting parameters of midostaurin (MIDO) concentration–response in SU-DIPG-50 cells after 48 h and 72 h incubation times in 96-well crystal violet (CV) and CCK-8 assays.

SU- DIPG-50 Cells	MIDO CV			MIDO CCK-8		
	IC_50_ (μM)	Hill Slope	Emax (%)	IC_50_ (μM)	Hill Slope	Emax (%)
48 h	0.86 μM	−6.9	−100%	0.12 μM	−7.9	−100%
72 h	0.13 μM	−8.3	−100%	1.4 nM	−11.8	−100%

**Table 5:** Changes in percentage (%) of CTRL current, after the application of MIDO at t = 0 and after 5 and 20 min of incubation in SU-DIPG-50 cells.

Incubation Times (min)	MIDO (%) at +60 mV (Vm)	MIDO (%) at −60 mV (Vm)
0	0	0
5	−84 ± 8	−40 ± 7
20	−100	−100

**Table 6:** Changes in percentage (%) of membrane potential, after the application of REPA, MIDO and BaCl_2_ + TEA at 4 different membrane voltages: −80, −60, +60 and +80 mV in SU-DIPG-50 cells.

Voltage Membrane (mV)	REPA (%)	MIDO (%)	BaCl_2_ + TEA (%)
−80	−15.2 ± 12	−11.69 ± 8	−9.92 ± 4
−60	−3.89 ± 1	−6.0 ± 3	−2.4 ± 1
+60	−27.62 ± 14	−35.72 ± 9	−78.78 ± 11
+80	−13.78 ± 21	−24.77 ± 8	−99.4 ± 1

## Data Availability

Data is available on request.

## Supplementary Materials

The following supporting information can be downloaded at: https://www.mdpi.com/article/10.3390/cancers18071066/s1, Table S1: Antibody list for Western blotting; Figures S1–S17: Original Western blot files.

## Abbreviations

The following abbreviations are used in this manuscript:

TKI	Tyrosine kinase inhibitor
MIDO	Midostaurin
STS	Staurosporine
DASA	Dasatinib
EVE	Everolimus
CRIZO	Crizotinib
LAPA	Lapatinib
ERLO	Erlotinib
PERIF	Perifosine
BaCl_2_	Barium chloride
TEA	Tetraethylammonium hydrochloride
CAPSZ	Capsazepine
RR	Ruthenium red
VEGFR2	Vascular endothelial growth factor receptor 2
mTOR	Mammalian target of rapamycin
PDGFR	Platelet-derived growth factor receptor
LC3	Microtubule-associated protein 1A/1B-light chain
AKT	Serine/threonine protein kinase
GLIB	Glibenclamide
REPA	Repaglinide
K-ATP	ATP-sensitive potassium channel
TRP	Transient receptor potential
