# Supplementary material for: Actions of Midostaurin as Cation Channel and Tyrosine Kinase Inhibitor in Diffuse Intrinsic Pontine Glioma Cell Lines
Source: Cancers (Basel). 2026 Mar 25;18(7):1066. doi: 10.3390/cancers18071066 (PMC13072310; doi:10.3390/cancers18071066)
Supplement: Supplementary file 1 [file cancers-18-01066-s001.zip › cancers-4180180-supplementary.pdf]

Supplementary Materials: Actions of Midostaurin as Cation Channel and Tyrosine Kinase Inhibitor in Diffuse Intrinsic Pontine Glioma Cell Lines

Marina Antonacci, Annamaria Di Turi, Morena Miciaccia, Michele Denora, Fatima Maqoud, Maria Grazia Perrone, Antonio Scilimati and Domenico Tricarico

Table S1. Antibody list for Western blotting.

Protein detected	Antibody	Source
mTOR (7C10)	2983	Cell Signaling Technology
pmTOR (Ser2448)	2971	Cell Signaling Technology
AKT pan (11E7)	4685	Cell Signaling Technology
Pakt (Ser473) (D9E)	4060	Cell Signaling Technology
Casp3 (3G2)	9668	Cell Signaling Technology
Claeved Casp3 (Asp175, 5A1E)	9664	Cell Signaling Technology
p44/42 MAPK (ERK1/2)(L34F12)	4696	Cell Signaling Technology
pERK1/2 (Thr202/Tyr204) (197G2)	4377	Cell Signaling Technology
Acetyl-Histone H3 (Lys27) H3K27ac	720096	Invitrogen
Histone H3 D1H2 XP	4499	Cell Signaling Technology
Try-Methyl-Histone H3 Lys 27	9733	Cell Signaling Technology

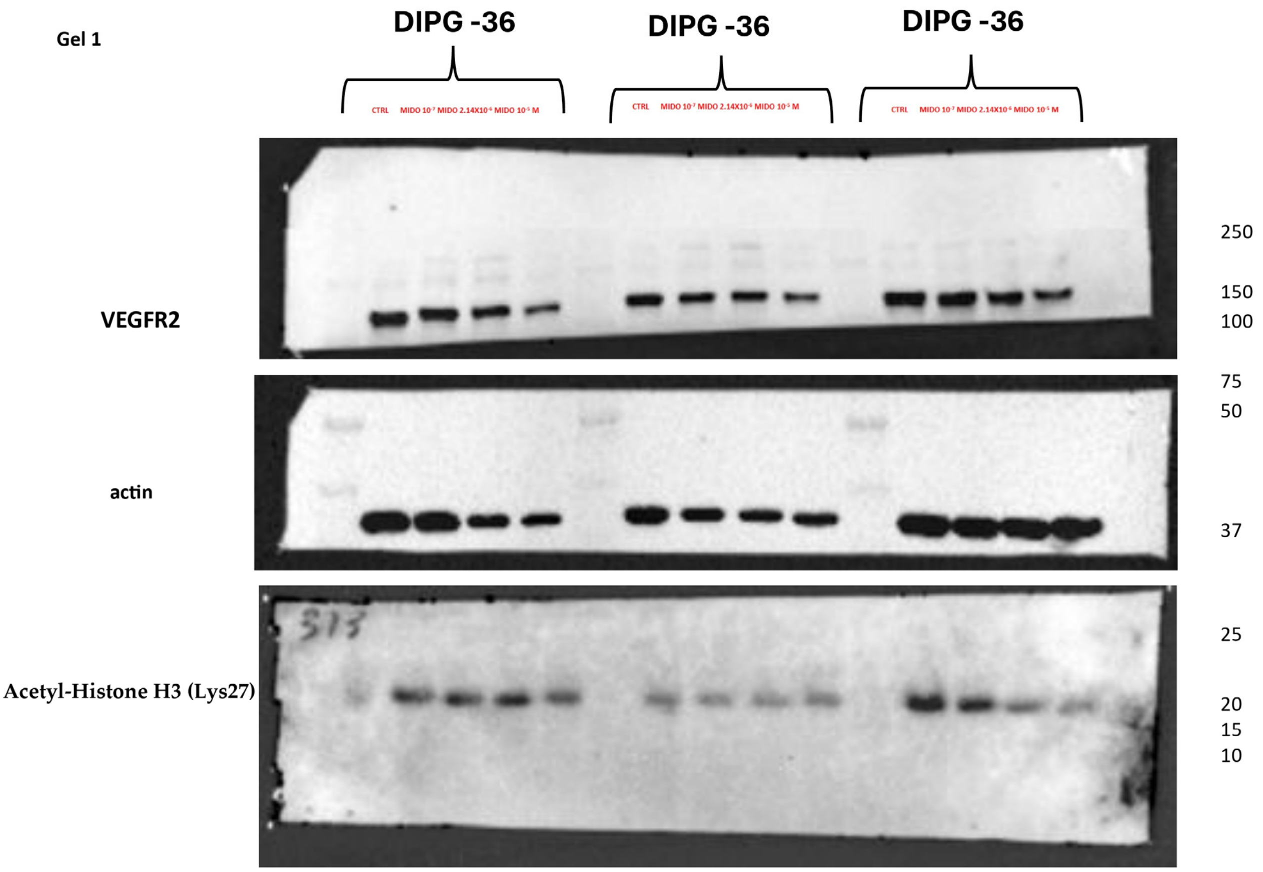

Figure S1. Original Western blot files.

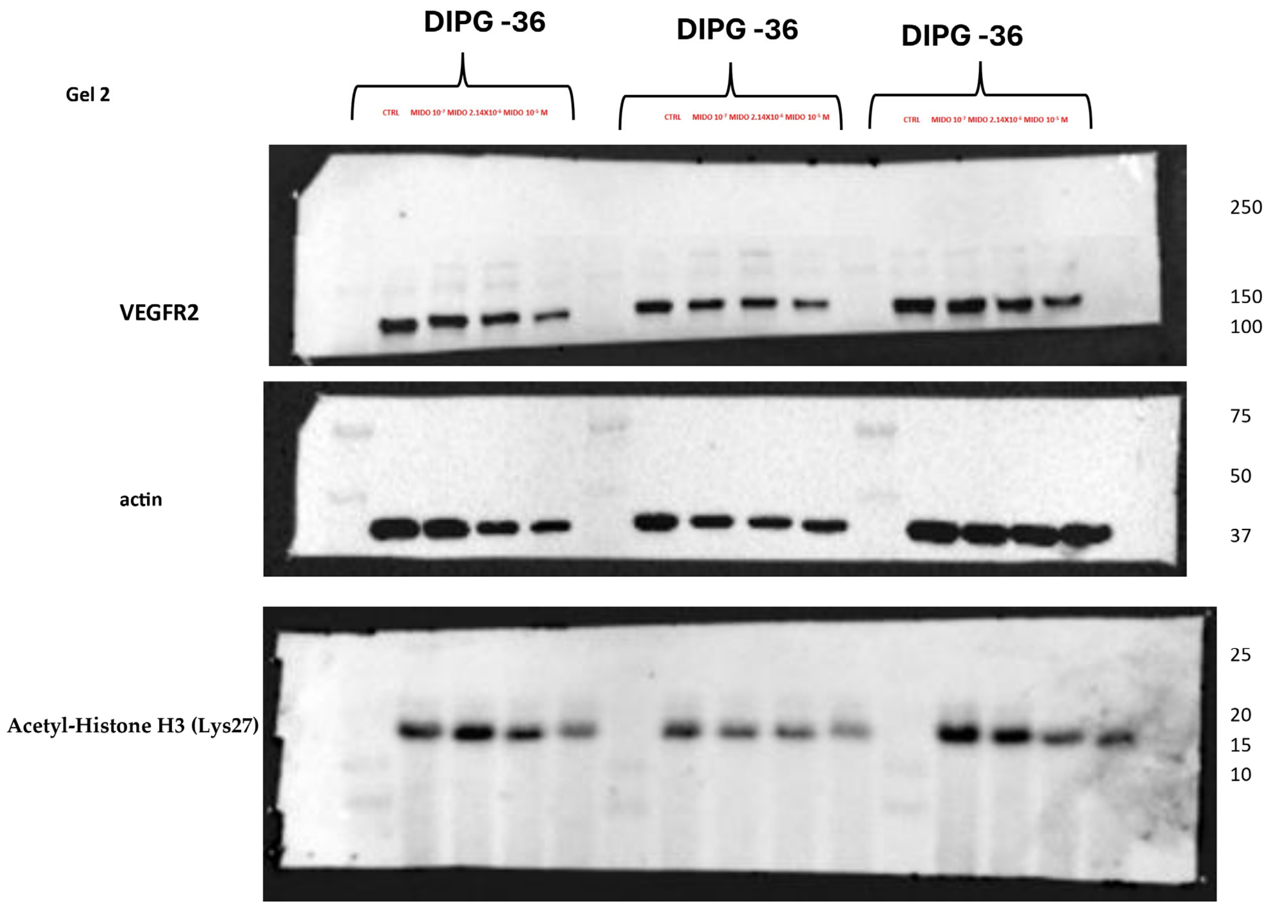

Figure S2. Original Western blot files.

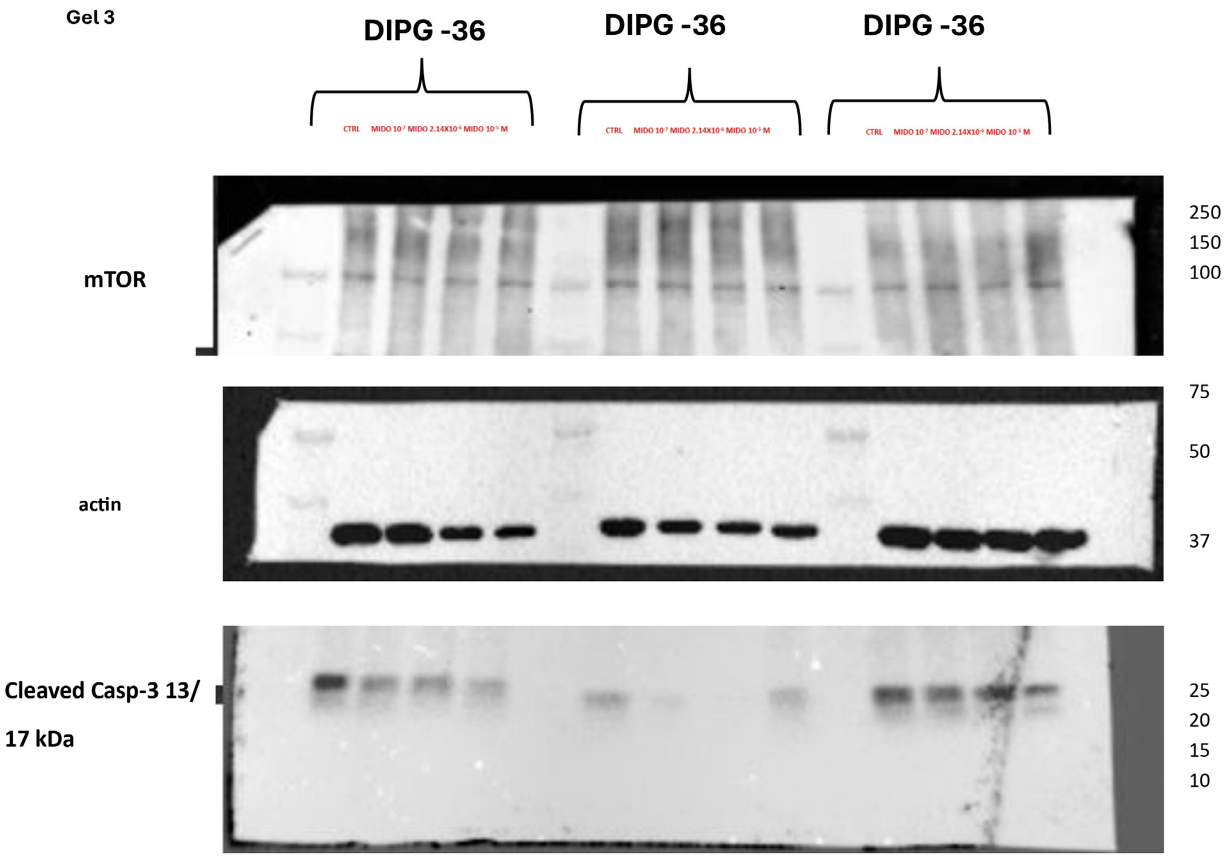

Figure S3. Original Western blot files.

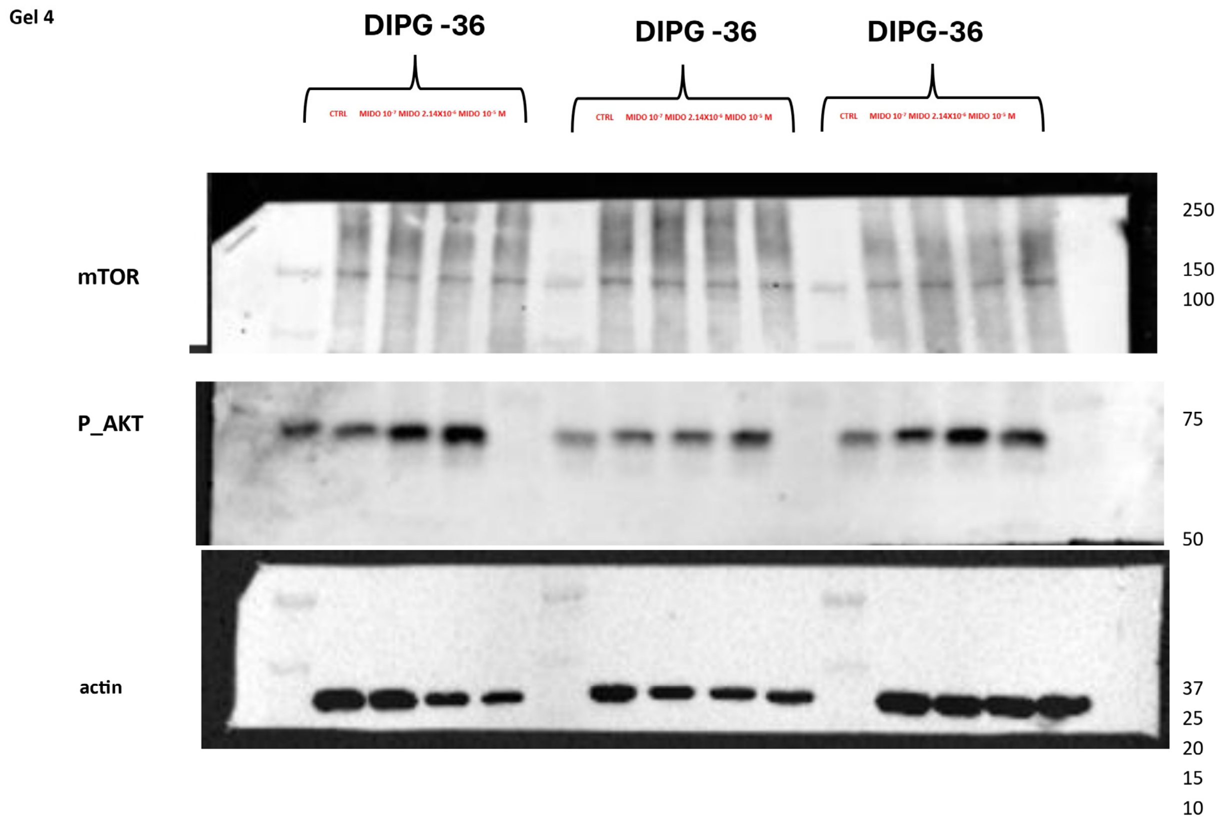

Figure S4. Original Western blot files.

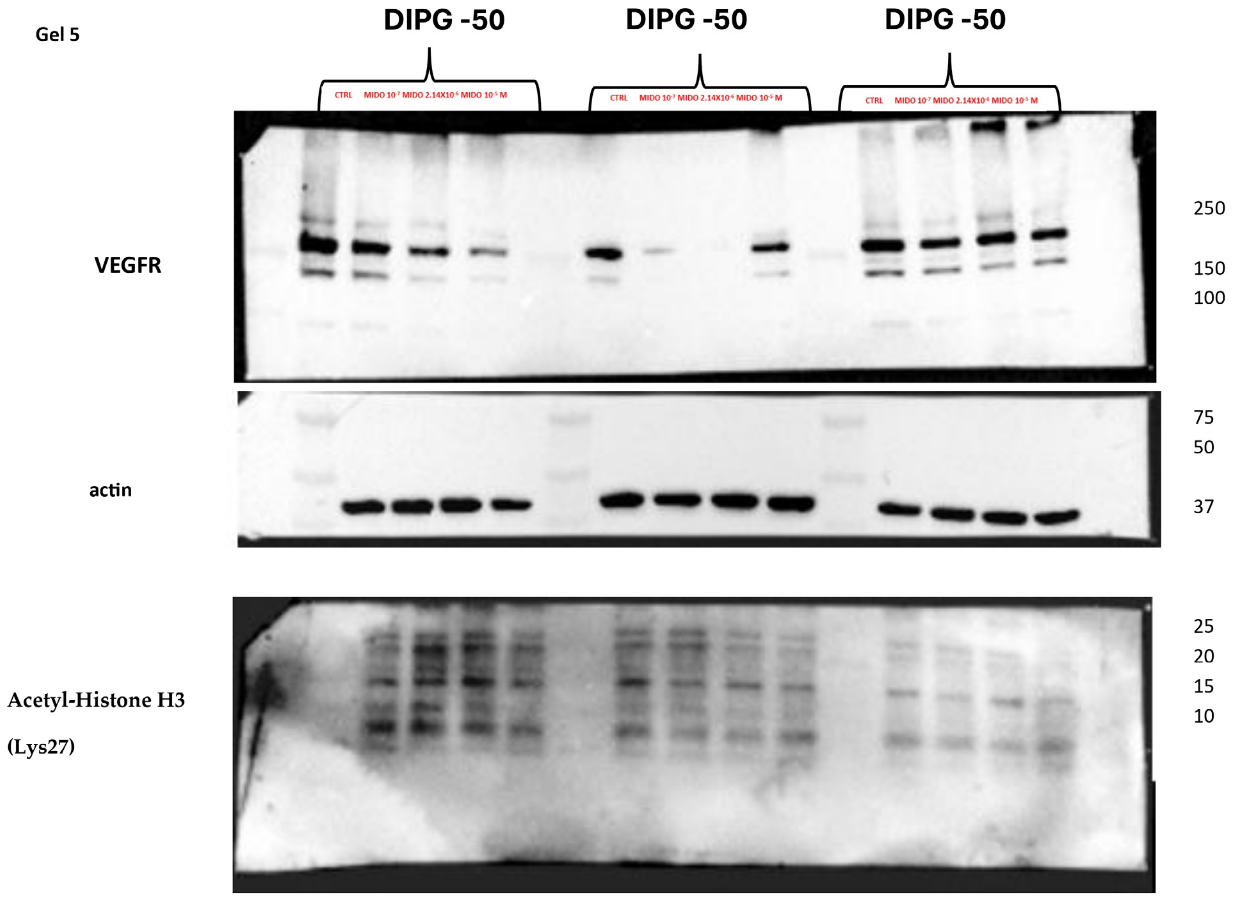

Figure S5. Original Western blot files.

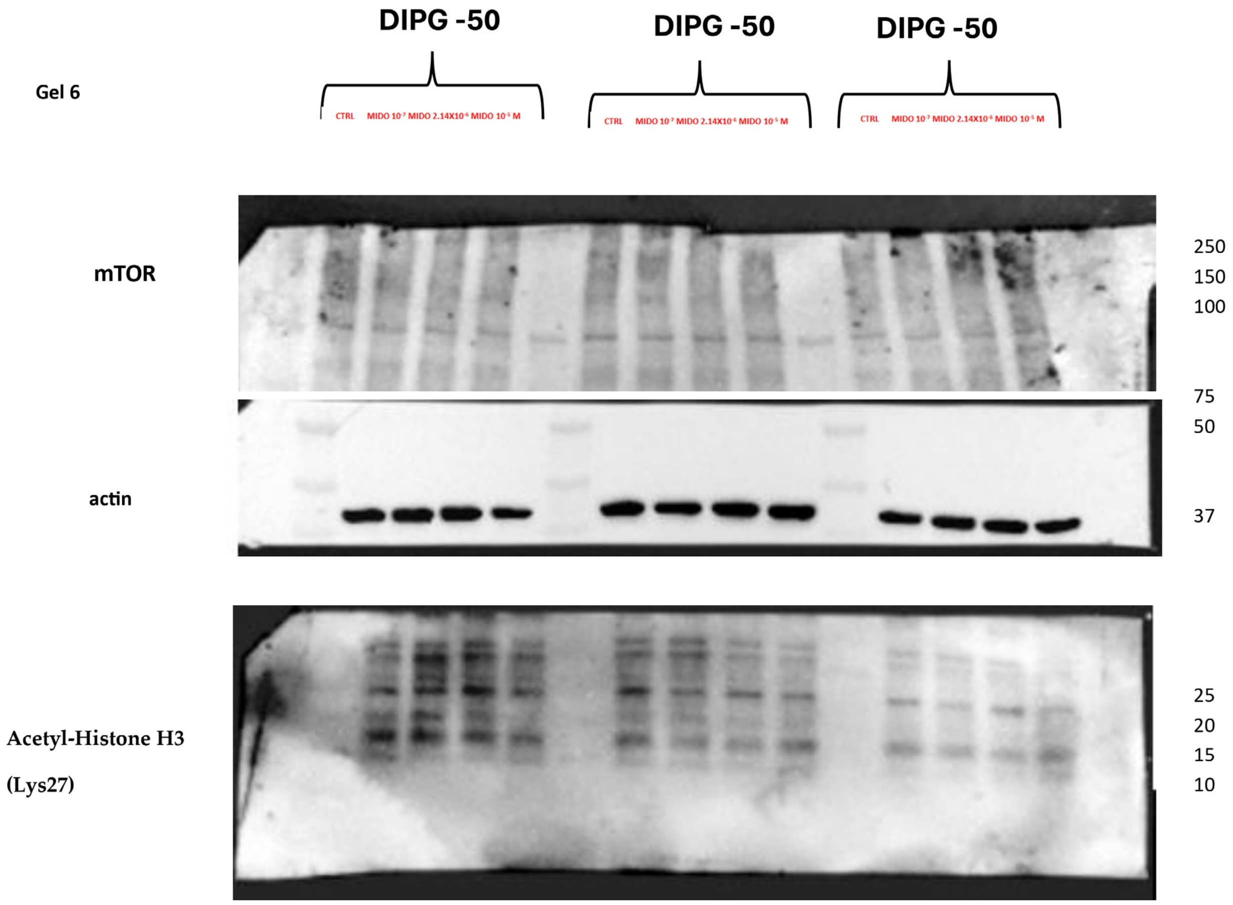

Figure S6. Original Western blot files.

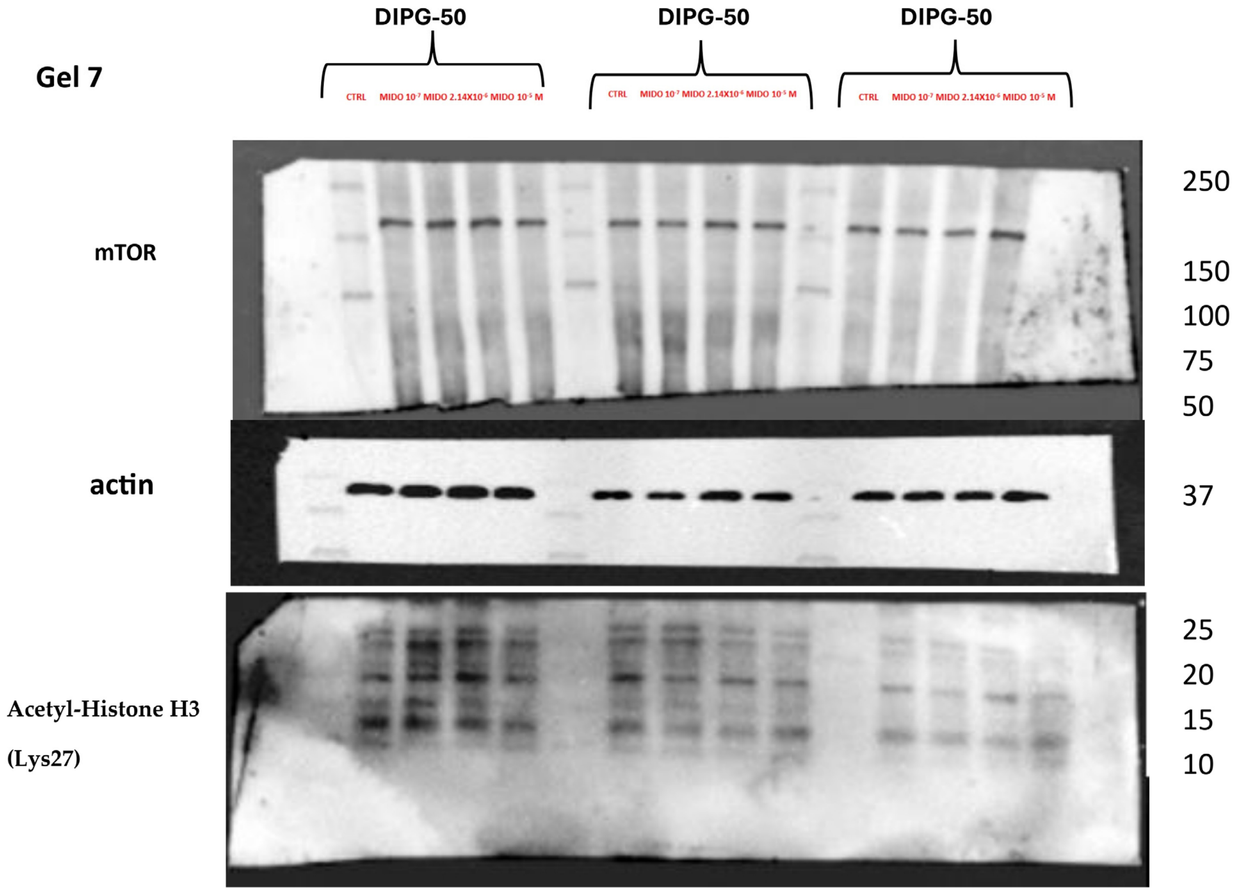

Figure S7. Original Western blot files.

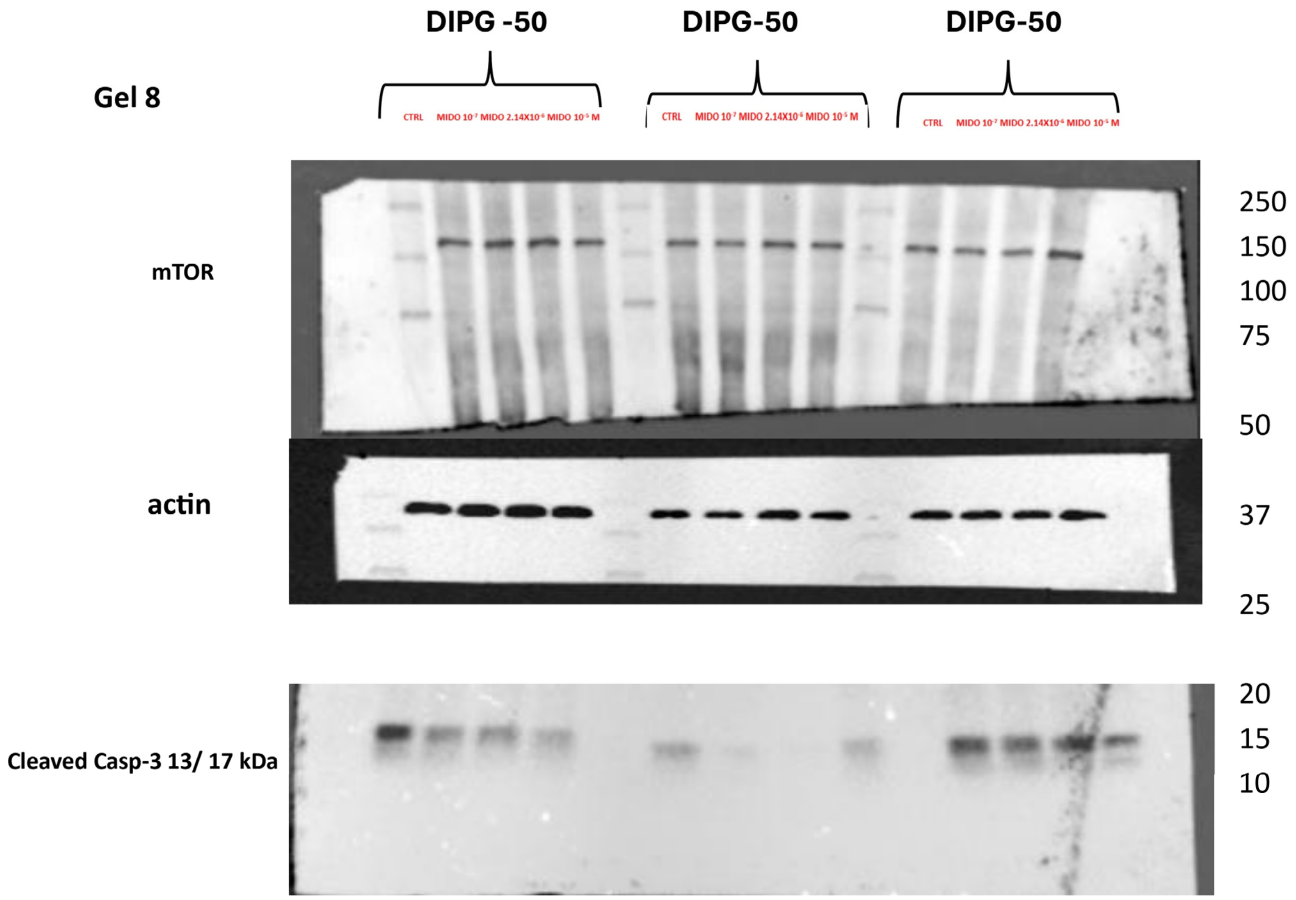

Figure S8. Original Western blot files.

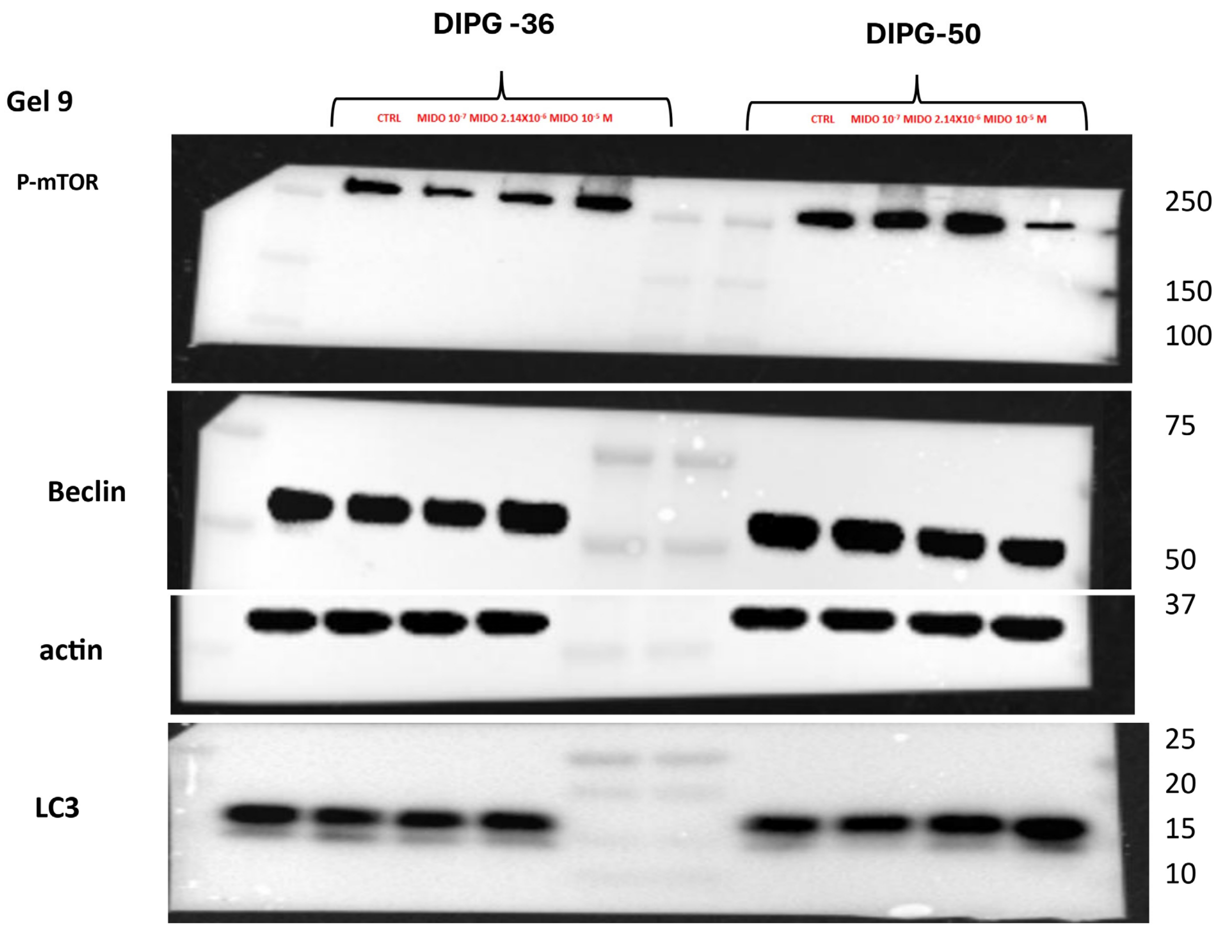

Figure S9. Original Western blot files.

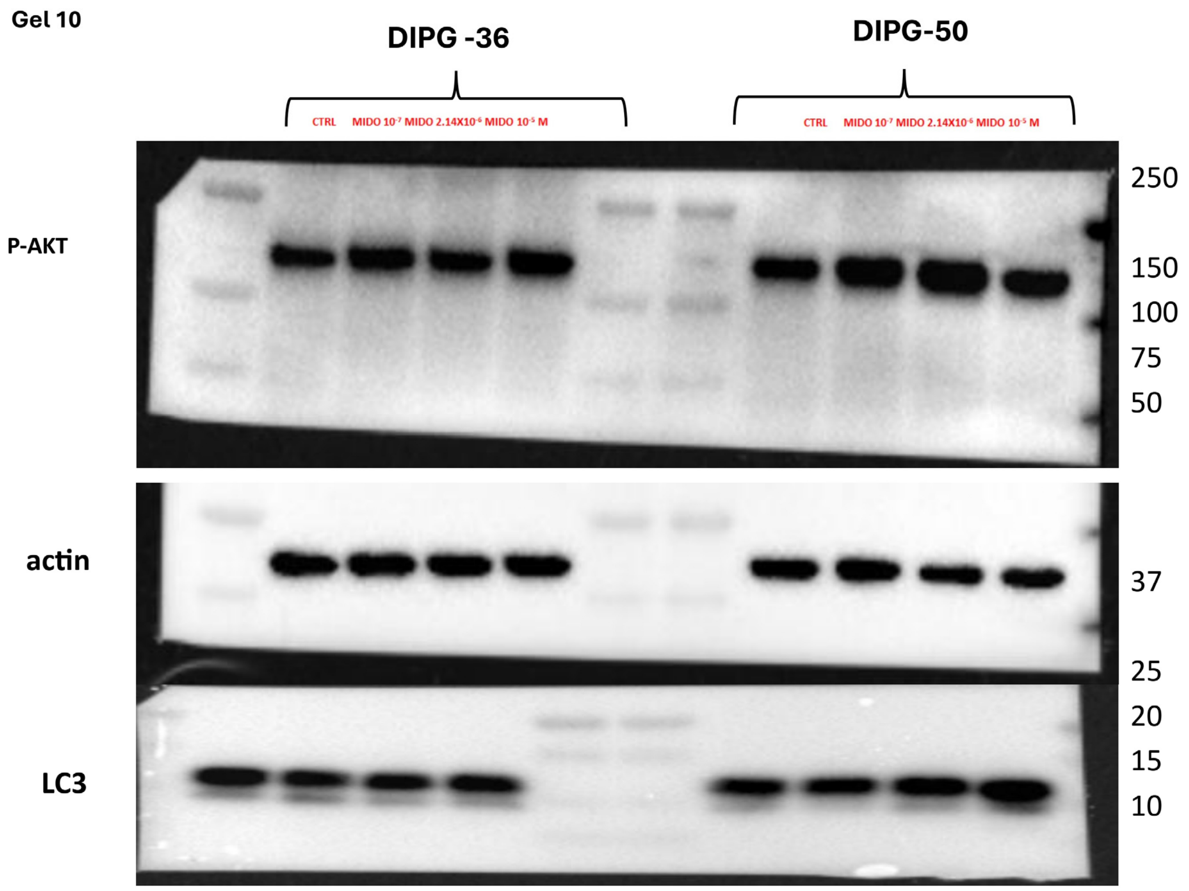

Figure S10. Original Western blot files.

https://doi.org/10.3390/cancers18071066

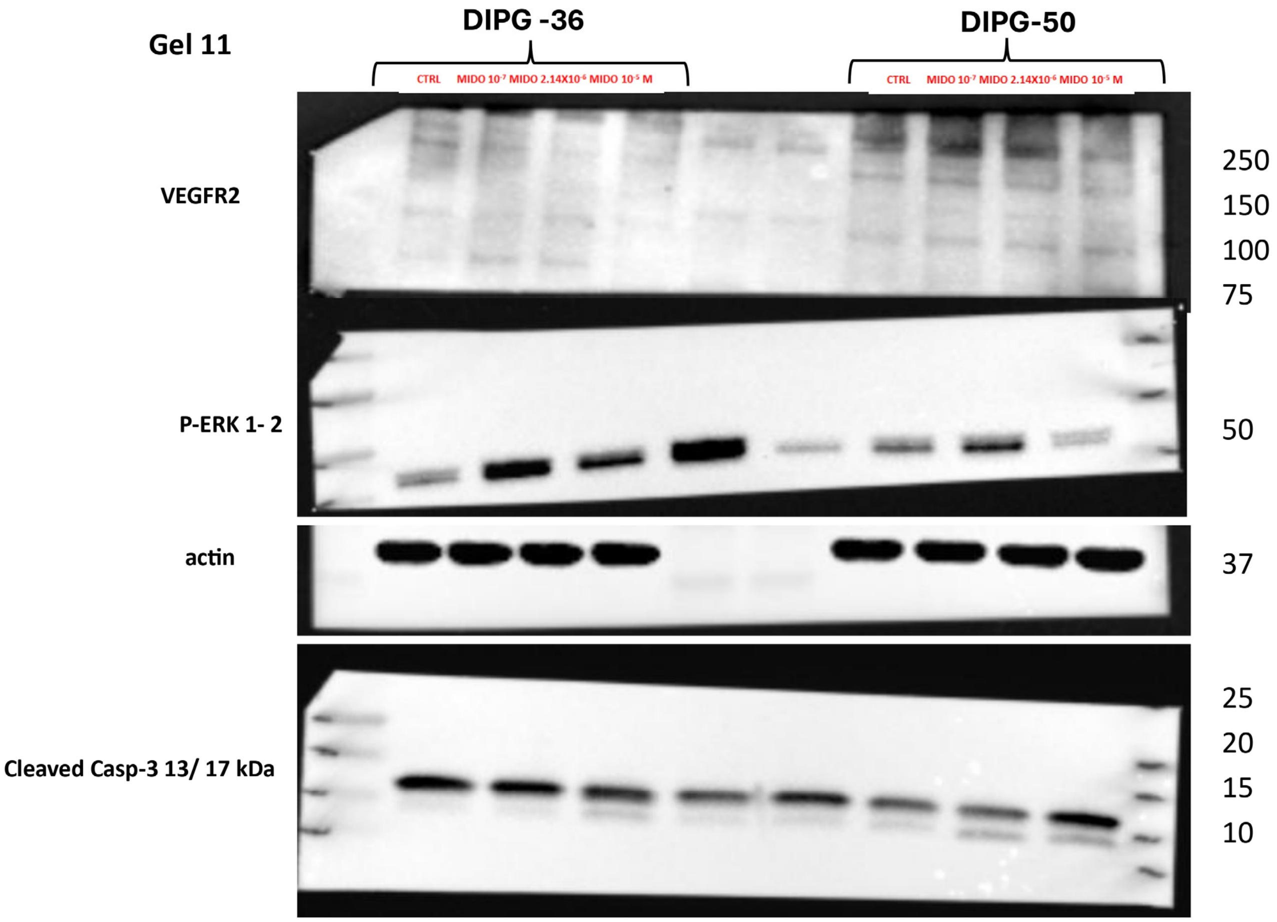

Figure S11. Original Western blot files.

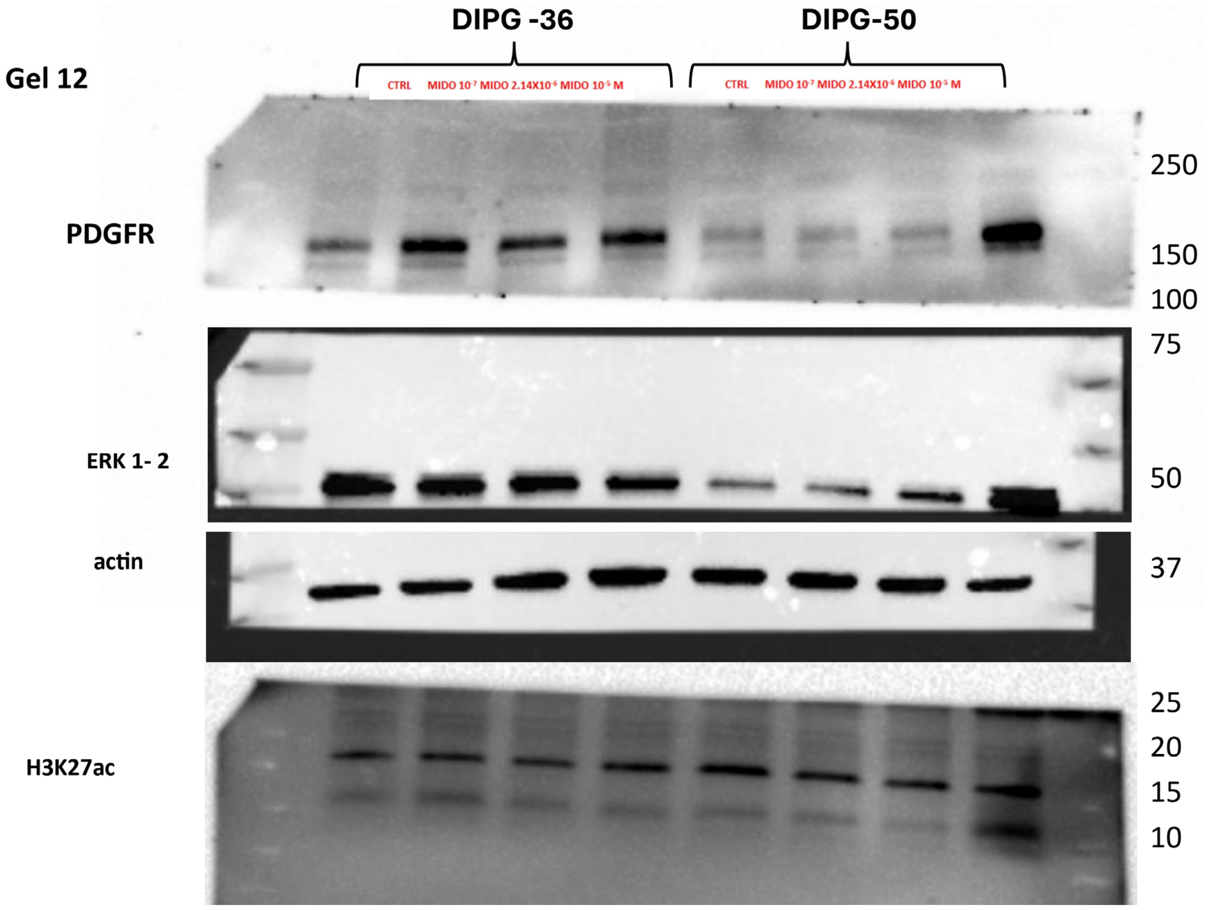

Figure S12. Original Western blot files.

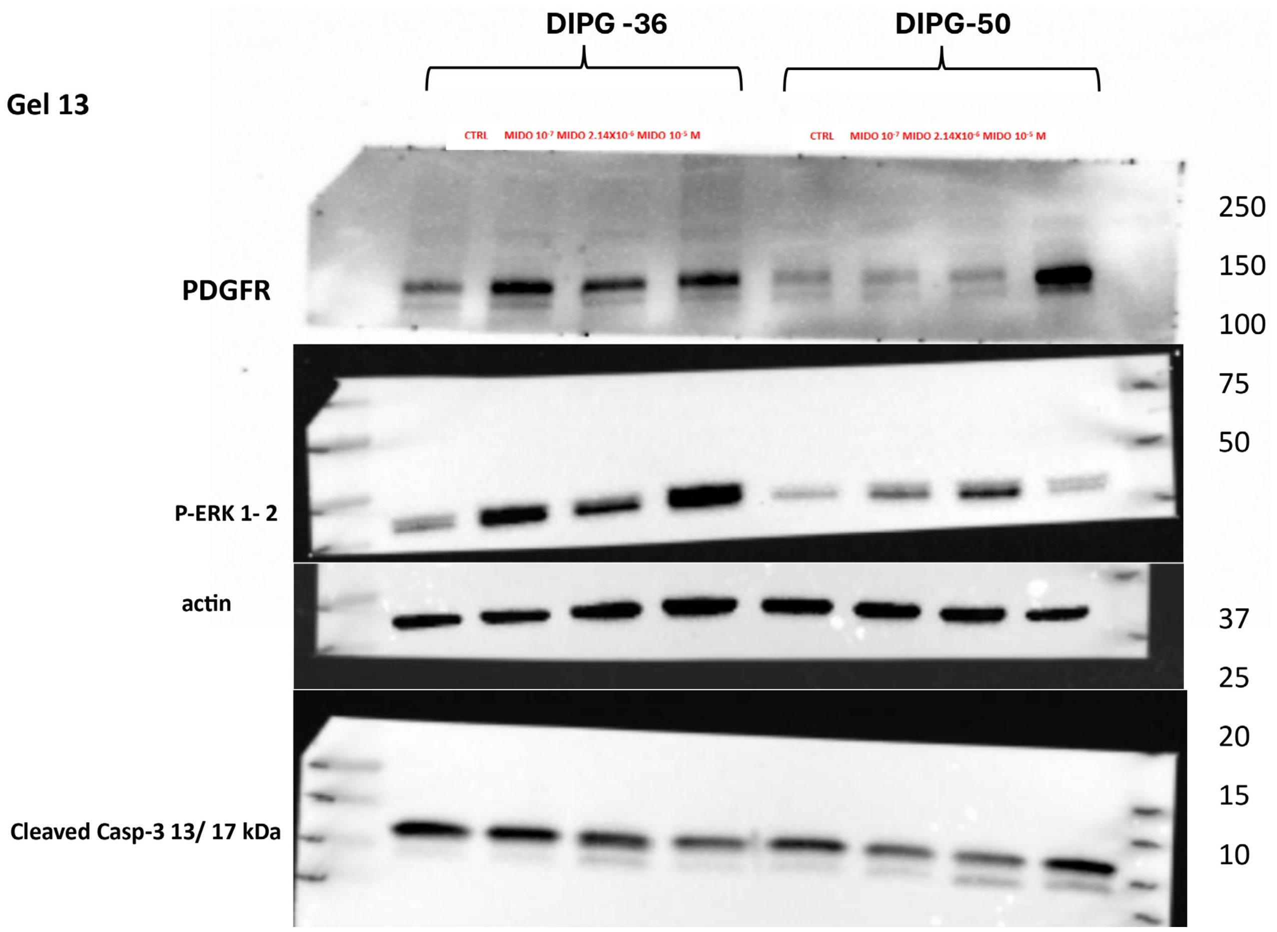

Figure S13. Original Western blot files.

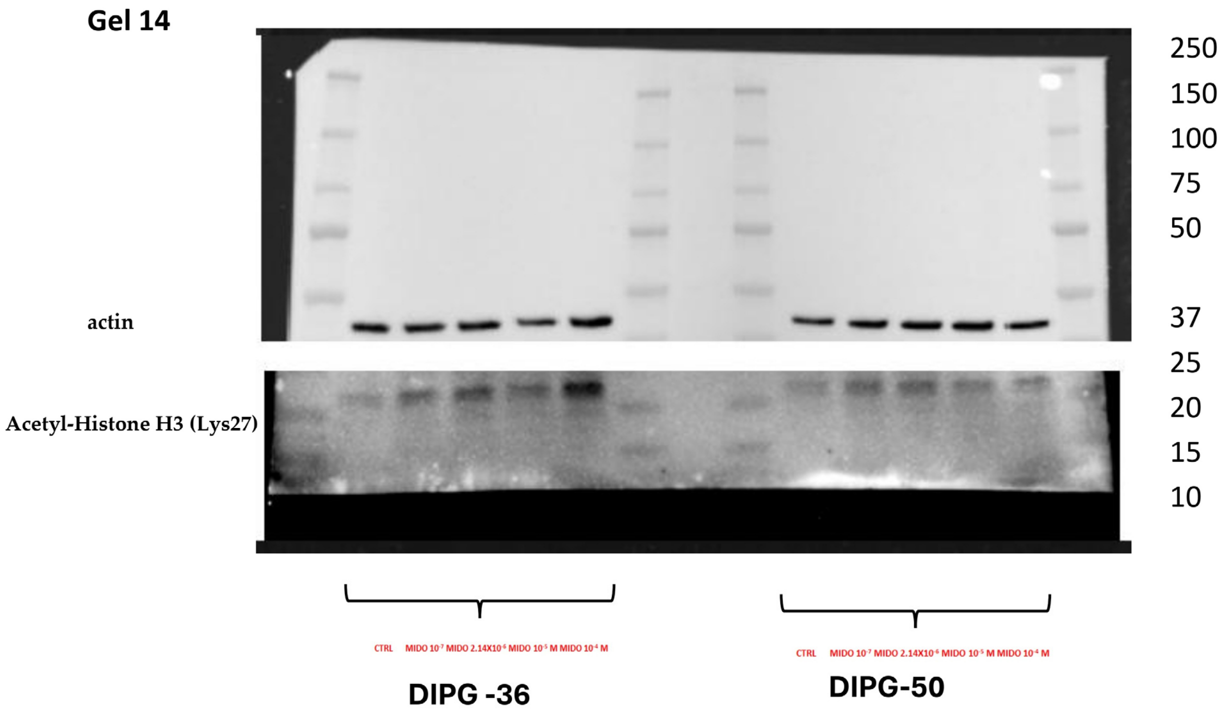

Figure S14. Original Western blot files.

https://doi.org/10.3390/cancers18071066

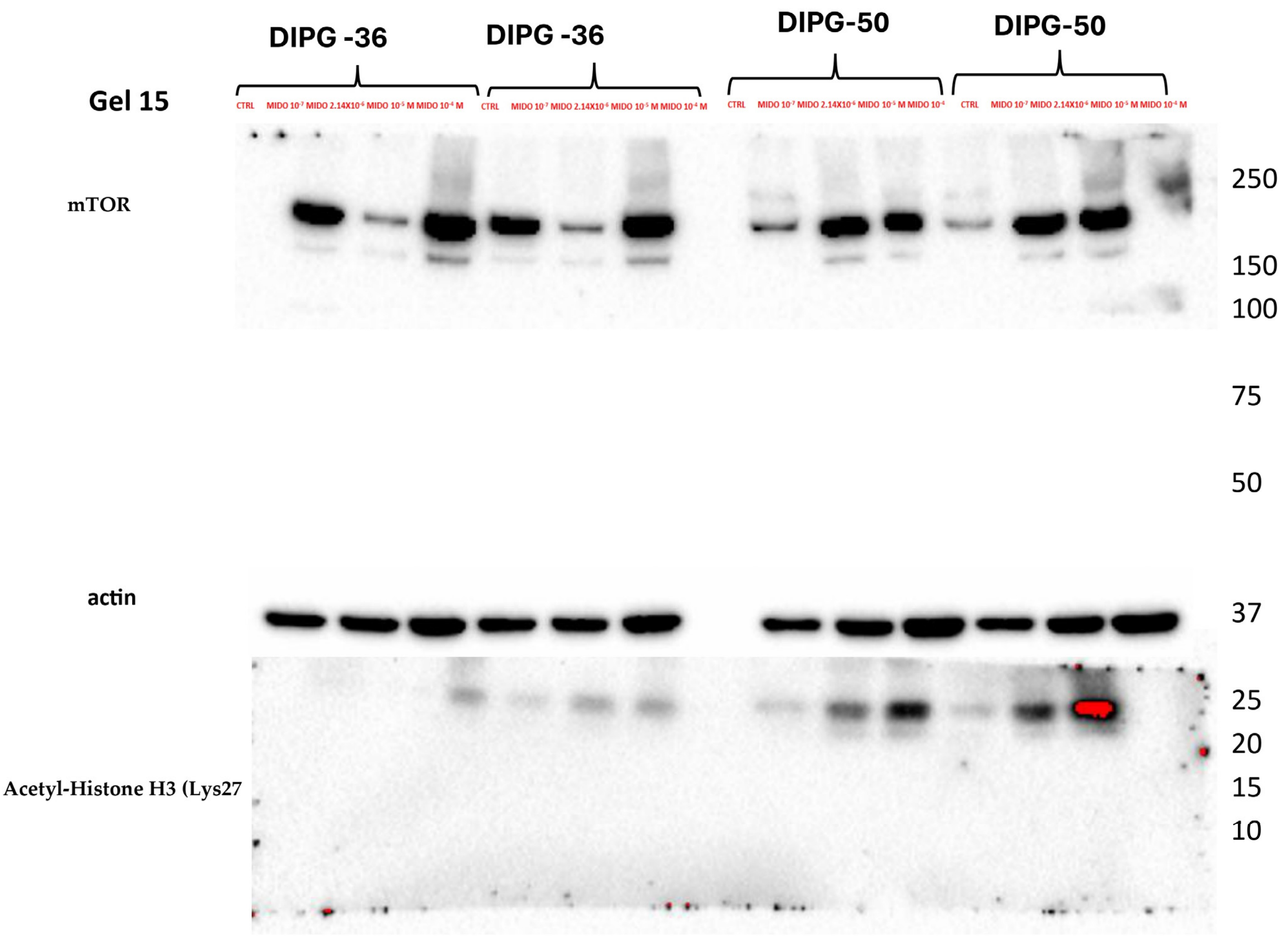

Figure S15. Original Western blot files.

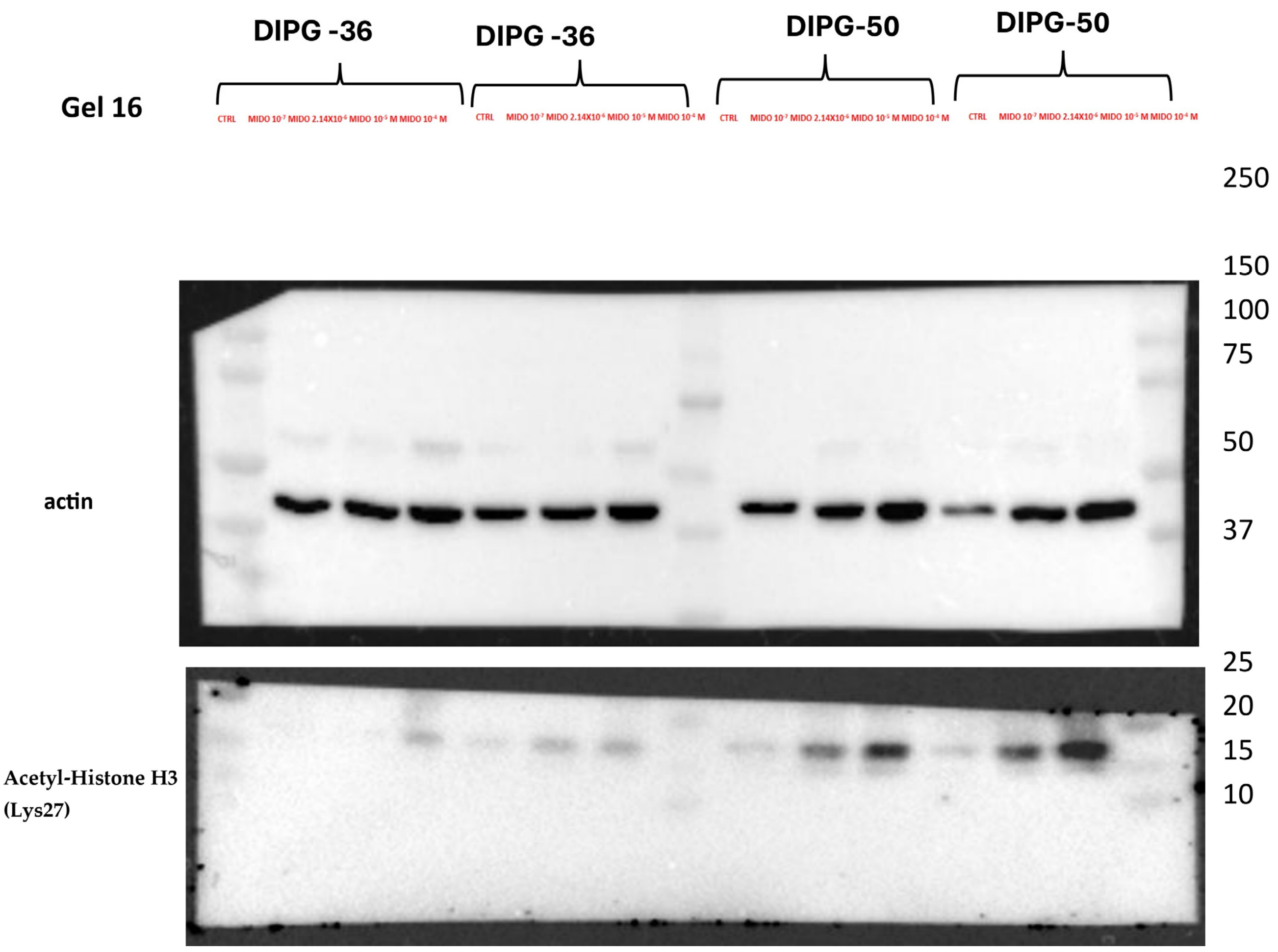

Figure S16. Original Western blot files.

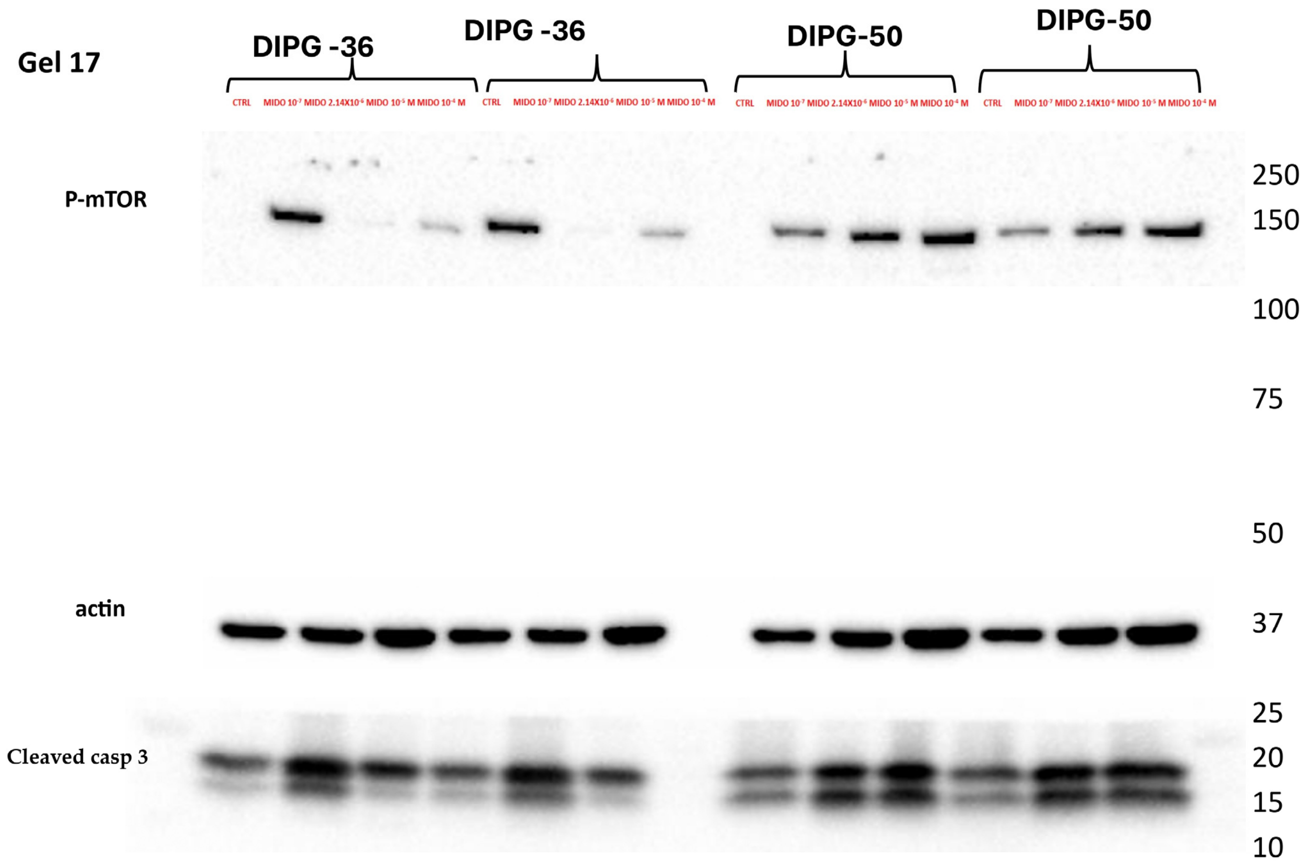

Figure S17. Original Western blot files.

https://doi.org/10.3390/cancers18071066
